# Therapeutics to Treat Psychiatric and Neurological Disorders: A Promising Perspective from Algerian Traditional Medicine

**DOI:** 10.3390/plants12223860

**Published:** 2023-11-15

**Authors:** Farida Larit, Francisco León

**Affiliations:** 1 Laboratoire d’Obtention de Substances Thérapeutiques (LOST), Université Frères Mentouri-Constantine 1, Route de Ain El Bey, Constantine 25017, Algeria; 2 Department of Drug Discovery and Biomedical Sciences, College of Pharmacy, University of South Carolina, Columbia, SC 29208, USA; jleon@mailbox.sc.edu

**Keywords:** Algeria, medicinal plants, CNS, psychiatric, neurological disorders, ethnobotanical, traditional healers, traditional medicine, pharmacopoeia, Africa, Chinese medicine, Ayurveda

## Abstract

Ancient people sought out drugs in nature to prevent, cure, and treat their diseases, including mental illnesses. Plants were their primary source for meeting their healthcare needs. In Algeria, folk medicine remains a fundamental part of the local intangible knowledge. This study aims to conduct a comprehensive ethnomedicinal investigation and documentation of medicinal plants and the different plant formulations traditionally used in Algeria for the treatment of pain, psychiatric, and neurological disorders. It also intends to improve the current knowledge of Algerian folk medicine. Several scientific databases were used to accomplish this work. Based on this investigation, we identified 82 plant species belonging to 69 genera and spanning 38 distinct botanical families used as remedies to treat various psychological and neurological conditions. Their traditional uses and methods of preparation, along with their phytochemical composition, main bioactive constituents, and toxicity were noted. Therefore, this review provides a new resource of information on Algerian medicinal plants used in the treatment and management of neurological and psychological diseases, which can be useful not only for the documentation and conservation of traditional knowledge, but also for conducting future phytochemical and pharmacological studies.

## 1. Introduction

**Figure 1:**
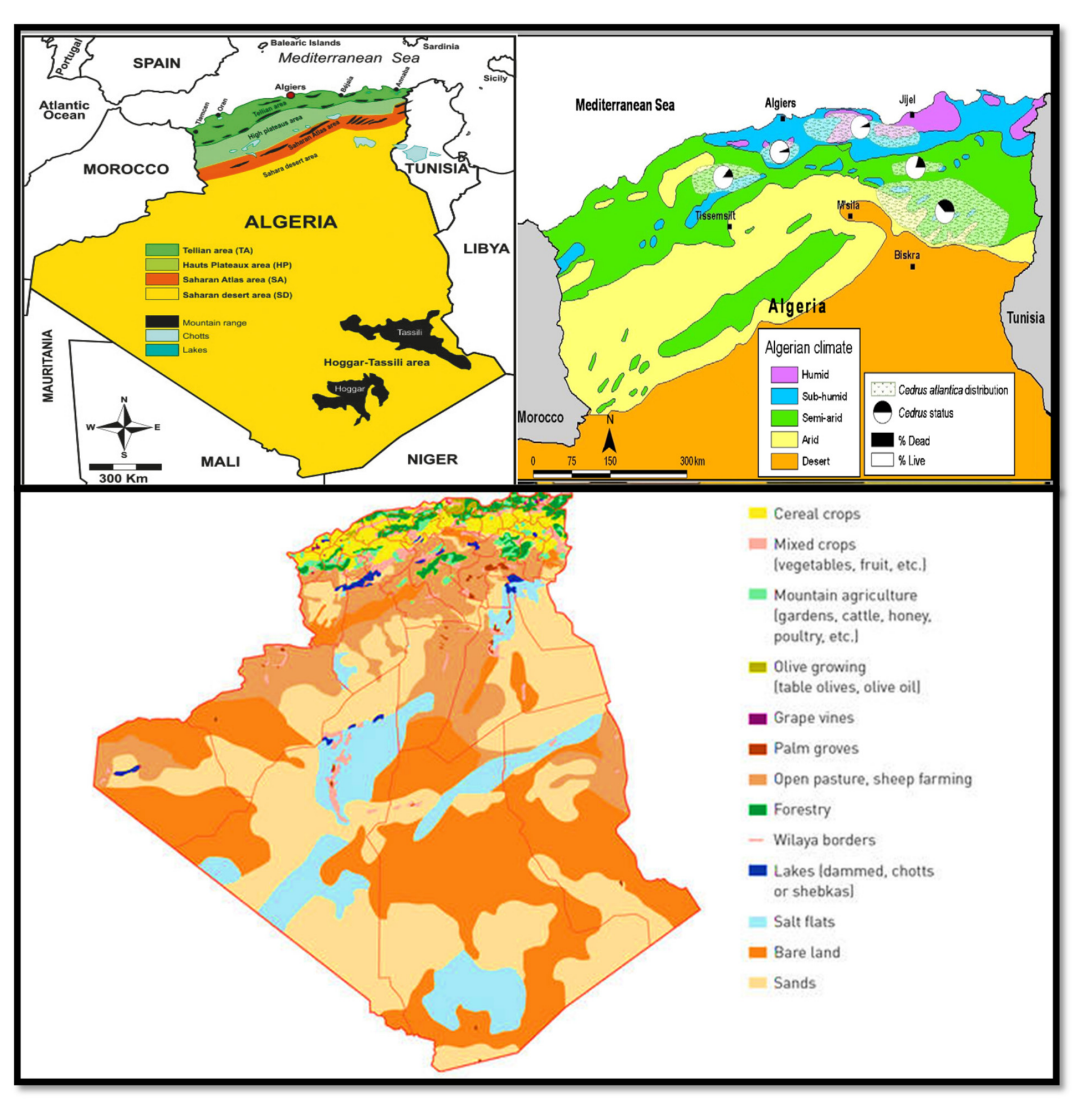
Major biogeographical/bioclimatic regions of Algeria [6,7,8].

Algeria is the largest country in Africa. It has a diverse range of landforms, including a Mediterranean coast that is separated from the Sahara Desert by a highlands plateau (Figure 1). Furthermore, Algeria experiences a diverse range of climates, spanning from a mild Mediterranean climate along the coast to highland winters marked by snowfall and desert regions characterized by scorching heat. This unique geographical positioning and climatic diversity have fostered the growth of a remarkably diverse flora encompassing an assortment of trees, shrubs, and herbs. Throughout ancient history, this rich and varied flora has consistently served as a primary source of raw materials for traditional medicinal practices [1,2,3,4,5].

Folk medicine comprises a reservoir of knowledge and practices utilized for the preservation of health and the prevention, diagnosis, enhancement, or treatment of both physical and mental ailments. This shared ethnopharmacological heritage has been transmitted across generations for millennia through both oral traditions and written records.

**Figure 2:**
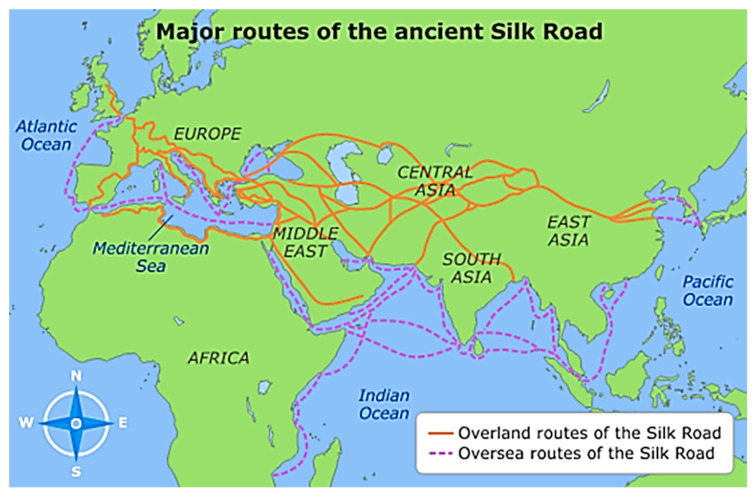
Routes of the Silk Road [16].

Indeed, Algerian ethnopharmacology predominantly represents a fusion of time-honored insights inherited from Islamic civilization and the pragmatic, empirical application of a diverse spectrum of substances, encompassing those originating from plants, animals, and inorganic sources [9]. For many centuries, the Islamic world was a center of scientific and medical enlightenment. This was due in part to the extensive network of trade routes that connected the East and West, including the famous Silk Road [10,11]. These routes facilitated the exchange of ideas, knowledge, and goods, allowing for the spread of scientific and medical advancements throughout the region (Figure 2). New disciplines emerged—algebra, trigonometry, and chemistry—as well as major advances in medicine, astronomy, engineering, and agriculture. Arabic texts replaced Greek as the fonts of wisdom [12]. Through their interactions with Asian traditions, including Chinese and Hindu influences, Arab pharmacists ingeniously merged medicinal plant knowledge, harmonizing their effects, and enhancing their palatability. Furthermore, indigenous medical wisdom, encompassing traditions, like traditional Chinese medicine, Ayurveda, and Tibetan practices, disseminated the essence of healing globally [10]. Islamic intellectual heritage has recognized mental health and played an active role in cultivating mental well-being, starting from the Prophet and inspiring Muslim scholars who followed. The Holy Quran is also considered a source of healing and comfort for mental and physical ailments [13,14]. Traditional Arabic and Islamic medicines are based on the concept of restoring balance to the body through diet, lifestyle, exercise, body cleansing, and the health of the mind, body, and spirit [15]. In Algeria, it is considered a form of alternative medicine that focuses on preventing and treating health conditions using methods and theories derived from the Holy Quran and Hadiths (Prophetic Medicine). These practices incorporate herbal medicines, spiritual therapies, dietary practices, mind–body practices, and manual techniques applied singularly or in combination to treat, diagnose, and prevent illnesses [15].

This review seeks to create an extensive catalog of medicinal plants available in Algeria, with a well-documented historical usage for treating, preventing, and managing mental and neuropsychiatric disorders. Additionally, it strives to provide a thorough insight into the potential therapeutic benefits linked to these plant species, serving as a foundation for guiding and motivating future phytochemical and pharmacological investigations.

## 2. Medicinal Plants Used for Mental Illnesses

Nervous system disorders include depressive disorders, anxiety disorders, bipolar disorder, schizophrenia, autism spectrum disorders, conduct disorder, attention-deficit hyperactivity disorder, eating disorders, idiopathic developmental intellectual disability, central nervous system conditions, like Alzheimer’s disease (AD) or Parkinson’s disease (PD), and a range of other mental disorders. Neurological disorders affect people of all ages, genders, education levels, and income levels in every country [17]. AD is the most common neurodegenerative disorder, according to the World Health Organization (WHO), and is one of the top 10 causes of death worldwide. Mental and neurological disorders affect approximately 907 million people worldwide [18]. In 2019, the global population of individuals affected by AD and other forms of dementia dramatically increased by a staggering 160.84% compared to the figures recorded in 1990, which were approximately 51.62 million. The number of global fatalities due to AD and other dementias rose from 0.56 million in 1990 to 1.62 million in 2019, nearly tripling over the span of three decades [19].

Traditional healing practices are indeed very important. The effectiveness of traditional treatment in psychotherapy has been demonstrated [20]. For example, the antipsychotic drug reserpine, an alkaloid isolated from the dried root of the *Rauwolfia serpentina* species, revolutionized the treatment of schizophrenia [21,22]. Research on natural psychoactive compounds has provided a wealth of information to the field of neuroscience [23,24]. Several medicinal plants have been shown to have beneficial effects on AD, PD, and depression [25,26]. Prescribed as a mild antidepressant, commercially available products of *Hypericum perforatum* are among the best-selling dietary supplements and are considered one of the most successful and effective herbal products in the world [27]. Another example is Janssen’s Alzheimer’s drug, Razadyne^®^ ER (also called galantamine), an alkaloid that was originally isolated from the *Galanthus* species and later found in other Amaryllidaceae genera, including daffodil bulbs (*Narcissus* genus) [28].

## 3. Traditional Treatment of Mental Disorders in Algeria

Depression, anxiety (including panic attacks and panic disorder), schizophrenia, epilepsy, insomnia, migraines, headaches, and eating disorders are common mental illnesses in Algeria [29]. These illnesses are also impacted by the spread of drug addiction and alcoholism in Algerian society. In fact, the number of individuals struggling with a drug addiction in Algeria is estimated to exceed 300,000 [30]. In 2019, there were 983 deaths attributed to drug use disorders in Algeria, accounting for a rate of 2.28 per 100,000 of the population [29] Furthermore, neuropsychiatric disorders are estimated to contribute to 13.1% of the global burden of disease [29]. According to a prior study conducted in Algiers, the capital of Algeria, the prevalence of anxiety disorders was as high as 43%, with 13% of individuals exhibiting symptoms of post-traumatic stress disorder [30].

Mental and neuropsychiatric disorders result from the intricate interplay of biological, psychological, social, and cultural factors. In some of the most remote African regions, such as among the nomad Berbers who uphold ancient traditions, the process of healing relies on a mystical realm deeply ingrained within these communities [31]. In traditional Algerian society, possession by a djinn (spirit) is considered the most likely cause of disease. Therefore, healers use plants with magical properties to restore one’s health. Someone presenting with mental disorders or chronic pain is often believed to have been a victim of the evil eye (Ein) and/or magic (Sihr) [32]. These are popularly considered causes of both physical and mental illnesses, often motivated by envy and jealousy. The evil eye and sorcery are seen as sanctions against anyone who exceeds the limits set by society in terms of wealth, beauty, intelligence, and happiness [32]. In such cases, traditional healers play a crucial role in identifying and countering these spiritual afflictions, using their knowledge of herbal remedies and rituals to bring about healing and balance within the affected individuals [33].

**Figure 3:**
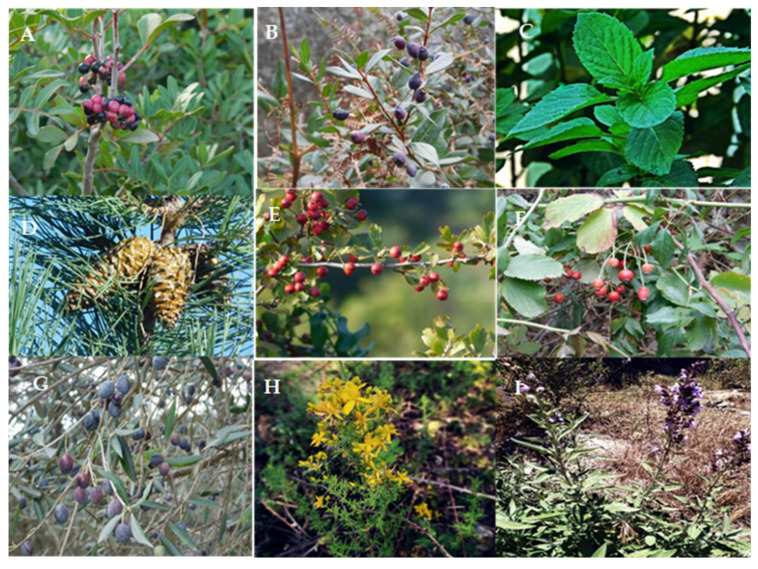
Algerian medicinal plants used to treat mental disorders. (**A**) *Pistacia lentiscus* L., (**B**) *Myrtus communis* L., (**C**) *Mentha piperita* L., (**D**) *Pinus halepensis* Mill., (**E**) *Crataegus oxyacantha* L., (**F**) *Crataegus azarolus* L., (**G**) *Olea europaea* L., (**H**) *Hypericum perforatum* L., (**I**) *Salvia officinalis* L.

The local population possesses valuable indigenous knowledge that has been passed down through generations [3,34,35,36]. Various plant parts, such as leaves, bark, fruit, buds, seeds, flowers, roots, and rhizomes, are employed for enhancing mental well-being and addressing various health issues [5,31]. Plant extracts are meticulously prepared through methods, like decoction, infusion, maceration, or tincture, and they can be consumed orally, applied topically, or administered through fumigation and vapor inhalation [31,34]. The historical use of numerous plant species has deep roots not only in Algeria but also in various other countries. Some, like *Atropa belladonna* (deadly nightshade) and *Ferula communis* L. (giant fennel), have associations with magic, while others, such as *Commiphora myrrha* (myrrh), hold significance in religious rituals [37]. Interestingly, species such as *Lavandula* spp. (lavender), *Marrubium vulgare* (white horehound), *Mentha* spp. (mint), *Ocimum basilicum* (basil), *Origanum majorana* (sweet marjoram), *Rosmarinus officinalis* (rosemary), *Salvia officinalis* (sage), *Stachys arvensis* (field woundwort), and *Thymus* spp. (thyme) were historically employed for protection against the evil eye [38] Decoctions made from *Stachys recta*, *Melissa officinalis*, or even *Myrtus communis* are traditionally employed for protection against negative influences or spirits [39] These herbal infusions have been historically and continue to be utilized for cleansing the face, hands, and wrists to alleviate various forms of pain, fear, and anxiety [39]. In Algeria, a rich diversity of 3139 plant species has been documented, of which 35% are from Mediterranean origins, with Euro-Mediterranean species accounting for 12% and North African species at 11%. Remarkably, 700 of these plant species are endemic [40]. The majority of plant species’ phytochemical and pharmacological properties remain undiscovered, and the realm of plant natural products remains largely unexplored. Only a handful of studies have explored the effects of Algerian medicinal plants on psychiatric and central nervous system disorders [41,42,43,44]. Figure 3 displays some common Algerian medicinal plants that have the potential to be used in the treatment of mental disorders.

## 4. Results

### 4.1. Medicinal Plant Diversity

The data for 82 plants traditionally utilized in the management of neurological and psychological disorders in Algeria were categorized based on their accepted Latin names, as per http://www.plantlist.org, and were grouped according to their respective botanical families (see Table 1). In the table, each plant entry includes information, such as the family name, common name(s), documented experimental evidence of activity (when available), the specific plant part(s) used, traditional methods of preparation, any identified active constituent(s) (if known), as well as the relevant data regarding interaction and toxicity studies.

In total, this study identified 82 plant species belonging to 69 genera and spanning 38 distinct botanical families (as outlined in Table 1).

### 4.2. Most Frequently Cited Plant Species

**Figure 4:**
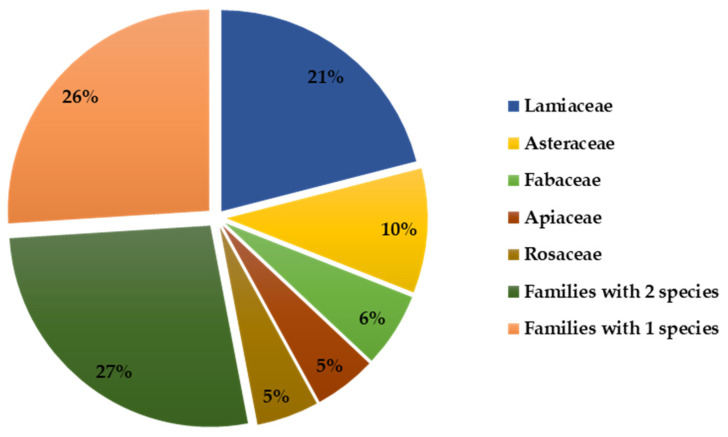
Distribution of the different reported species among the botanical families.

Many plants have been identified in ethnobotanical surveys, but only a limited number of pharmacological studies focusing on brain disorders in Algeria have been documented. This study uncovered a total of 82 medicinal species utilized for the treatment, management, and prevention of mental illnesses. These species are distributed among 69 genera and 38 families (refer to Table 1). The most frequently mentioned families included Lamiaceae, accounting for 17 species (21%), Asteraceae with 8 species (10%), Fabaceae with 5 species (6%), and both Apiaceae and Rosaceae, each comprising 4 species (5%). The remaining 33 families (53%) were represented by one to two species each (Figure 4).

*Mentha*, a genus within the Lamiaceae family, occupies a prominent position in the realm of botanical significance. With a substantial representation of 29% among genera of the Lamiaceae family (Table 1), it stands out as a noteworthy and extensively studied genus. The distinguishing characteristic of *Mentha* species lies in their aromatic nature, which is pervasive across various regions worldwide. It encompasses five distinct species: *Mentha arvensis* L., *Mentha aquatica* L., *Mentha rotundifolia* (L.) Huds., *Mentha pulegium* L., and *Mentha x piperita* L. Notably, peppermint (*Mentha x piperita* L.) is the most widely utilized among these species and stands as one of the most crucial commercially grown aromatic herbs globally. Peppermint essential oil possesses a distinctive sharp, cool, and invigorating aroma, and it carries significant pharmacological importance, primarily owing to its primary active component, menthol. Menthol exhibits a broad spectrum of biological activities and therapeutic potentials [115].

Peppermint is traditionally used in Algeria as a remedy for several conditions, including the common cold and headaches. It is also used as a sedative [34]. Its essential oil is commonly applied topically for relief from muscle pain and headaches. Many studies have shown that peppermint has a considerable effect on certain mental disorders, such as anxiety, stress, and insomnia [116,117]. It has been reported that the administration of a peppermint infusion can improve sleep quality and enhance memory [116].

Our results are consistent with previous studies that reports that the most commonly used plants in several Algerian regions belong mainly to the Lamiaceae, Asteraceae, and Apiaceae families [34,36,72]. A previous study conducted by A. Bouasla et al. revealed that *Mentha piperita* (Lamiaceae), *Thymus vulgaris* (Lamiaceae), *Zingiber officinale* (Zingiberaceae), *Myrtus communis* (Myrtaceae), *Nigella sativa* (Rutaceae), *Chamaemelum nobile* (Asteraceae), and *Olea europaea* (Oleaceae) were the species most frequently used to treat nervous system disorders in the region of Skikda in Northeastern Algeria [34].

### 4.3. Used Parts

It is evident that leaves are the most commonly utilized plant parts among local populations, not only in Algeria but also in numerous regions globally [36,118,119]. Our findings indicate that the most commonly employed plant parts are leaves, followed by the aerial parts, flowers, seeds, roots, and rhizomes. Additionally, other plant components, such as fruits, stems, barks, resin, capitulum, buds, and even the entire plant, were reported to be used, albeit at a lower frequency.

### 4.4. Method of Preparation

Our results unveil that the most prevalent methods for preparing plant remedies among local populations are decoction and infusion in water, consistent with the findings in other studies [34,36].

### 4.5. Pharmacognostic Investigations on the CNS System

The effects of medicinal plant preparations, their extracts, and isolated secondary metabolites on the central nervous system (CNS) have been extensively studied [120,121,122]. Numerous active principles from plants, such as polyphenols, polysaccharides, terpenoids, alkaloids, coumarins, tannins, and other natural bioactive ingredients, are used in the management of psychiatric and mental disorders [123,124,125]. For example, polyphenols, major groups of natural compounds that act as primary antioxidants [126,127], can protect DNA from oxidative damage, inhibit tumor cell growth, and possess anti-inflammatory and neuroprotective properties [128,129,130,131,132,133]. Flavonoids, including chalcones, flavones, flavonols, flavanones, isoflavonoids, anthocyanins, and proanthocyanidins, are widely distributed in the plant kingdom. These compounds exhibit various pharmacological effects, such as antioxidant, anti-inflammatory, anxiolytic, sedative, anticonvulsant, and analgesic properties, through their actions on the CNS [134]. Recent studies have shown that the regular consumption of flavonoid-rich foods can effectively enhance cognitive functions in humans [135,136,137]. Moreover, several flavonoids have been shown to prevent the progression of AD pathologies by reversing the cognitive deficits in various normal and transgenic animal models [135,137,138]. Essential oils are another example of active compounds derived from aromatic plants. Their primary constituents are vital agents well-known for their antimicrobial and antifungal properties, alongside their recognized roles in antiviral, antioxidant, anti-inflammatory, antidiabetic, and anticancer activities [139]. Several studies have demonstrated the relationship between the chemical composition and biological properties of essential oils and their applications in various commercial and pharmacological preparations to prevent, treat, and manage neurological disorders [140,141]. Moreover, numerous research works have proven that the inhalation of certain essential oils improves mood, enhances memory, and reduces stress [142,143,144].

### 4.6. In Vitro and In Vivo Pharmacological Evidence

Numerous plant crude extracts, fractions, and isolated secondary metabolites have undergone binding studies targeting CNS receptors, including dopamine, serotonin, cholinergic, GABA, opioids, and cannabinoid receptors [145]. Subsequently, a series of in vivo studies were conducted using appropriate animal models, primarily rodents, to obtain essential pharmacological and toxicological data before progressing to human clinical trials [146,147] These animal studies helped establish the basic safety and efficacy profiles of the substances under investigation, paving the way for further research on human subjects.

**Figure 5:**
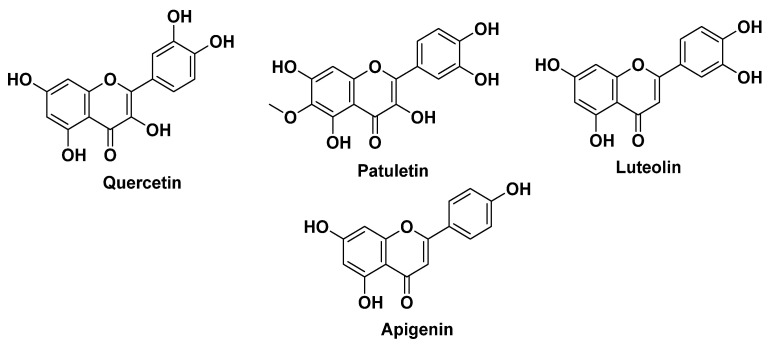
Selected flavonoids found in Roman chamomile.

Roman chamomile (*Chamaemelum nobile*, Asteraceae) finds extensive application in traditional medicine globally, with a notable presence in Algeria, particularly in the Northeastern region. *Chamaemelum nobile* is renowned for its tranquilizing properties, serving as a mild sedative to alleviate nervousness, anxiety, and a range of disorders. It has been historically employed to address conditions, such as hysteria, insomnia, and various ailments, earning its place as one of the oldest known medicinal herbs [34]. Flavonoids, including apigenin, luteolin, quercetin, and patuletin, constitute significant components in *Chamaemelum nobile* (Figure 5). Interestingly, these flavonoids are not exclusive to Roman chamomile; they are also present in other plants, like *Passiflora incarnata* (passionflower) and *Matricaria chamomilla* (chamomile) [31,62]. Apigenin has garnered attention due to its reported binding affinity to gamma-aminobutyric acid (GABA)-benzodiazepine receptors in the brain. This interaction with GABA receptors may be responsible for the sedative effects associated with apigenin [148]. Such insights into the specific bioactive compounds in *Chamaemelum nobile* contribute to our understanding of its potential pharmacological properties and its historical use as a natural remedy for calming and soothing effects [149].

*Artemisia absinthium* is a perennial herbaceous plant from the Asteraceae family, native to North Africa, including Algeria. Traditionally, it has been valued for its diverse therapeutic properties and widely used in conventional medicine across the world. It is employed for treating various conditions, such as digestive problems, morning sickness, irregular menstrual cycles, typhoid, epilepsy, renal issues, bronchitis, and malaria, among others [150]. Additionally, in Algerian traditional medicine, *Artemisia absinthium* is known to have calming properties that can potentially help with anxiety, restlessness, and insomnia [31,34,57]. In addition to flavonoids and phenolic acids, the plant also contains other compounds, one of which is thujone. Thujone, a volatile monoterpene ketone, has been shown to interact with GABA receptors in the brain [151]. This interaction suggests that thujone may exhibit GABAergic activity, which potentially underlies its sedative and anxiolytic effects [152].

*Valeriana officinalis* (Caprifoliaceae) is one of the most widely recognized herbal sedatives worldwide. It has long been used in alternative medicine for the treatment of insomnia and other related neurological disorders [153]. *Valeriana officinalis* is also cultivated in Algeria and traditionally used for treating neurological disorders [154]. The hydroalcoholic extract of *Valeriana officinalis* interacts with ionotropic glutamate receptors, believed to be one of the reasons for its anxiolytic effect [155]. Valerianic acid, a common standard marker for *Valeriana officinalis*, has also been observed to interact with metabolic glutamine receptors, which partly explains its anxiolytic effects [156]. The secondary metabolites of the *Valeriana* genus include, among others, sterols, alkaloids, and terpenes. Among the terpenes, valepotriates and sesquiterpenoids are noteworthy [157]. However, it is likely that the effects result from a summation and synergy of all essential oils, along with the terpenes found in *Valeriana officinalis* [158,159]. *Valeriana tuberosa* L., a Mediterranean species naturally found in Algeria, is traditionally considered as a complement in the prevention and treatment of epilepsy, as well as for its antispasmodic, calming, and hypotensive properties [46].

*Hypericum perforatum* (St. John’s wort) from the family of Hypericaceae stands out as one of the most extensively studied due to its recognized anti-depressant effects. In a study presented by Simmen et al. [160], *Hypericum perforatum* extracts, fractions, and constituents were subjected to radioligand binding assays on opioid, serotonin, estrogen, histamine, neurokinin, and metabotropic glutamate and GABA_A_ receptors. Hypericin exhibited the most pronounced binding inhibition with the human CRF1 receptor. Hyperforin hindered binding with opioid and serotonin (5-HT) receptors, while hypericin and pseudohypericin displayed comparatively weaker inhibitory effects. The biflavonoid I3,II8-biapigenin effectively restrained 3H-estradiol binding to the estrogen-alpha receptor. It is worth noting that the inhibition of 3H-muscimol binding to the GABA_A_ receptor is likely attributed solely to the presence of GABA in the extract. The study suggests that the beneficial antidepressant effect of St. John’s wort may result from the additive or synergistic actions of several distinct compounds [160].

In a previous investigation, Simmen et al. explored the impact of *Hypericum perforatum* extracts, fractions, and individual components on the binding of various ligands to recombinant CNS receptors expressed through the Semliki Forest virus expression system [161]. Among these, a lipophilic fraction of *Hypericum* exhibited notably strong inhibitions of mu-, delta-, and kappa-opioid receptors, as well as 5-HT6 and 5-HT7 receptors. Furthermore, constituents of Hypericum, including naphthodianthrones, like hypericin and pseudohypericin, and the phloroglucinol hyperforin, displayed inhibitory effects on both opioid and serotonin receptors in the lower micromolar range. These findings provide support for the notion that multiple active components in Hypericum may work synergistically to contribute to its antidepressant effects in the central nervous system [161].

**Figure 6:**
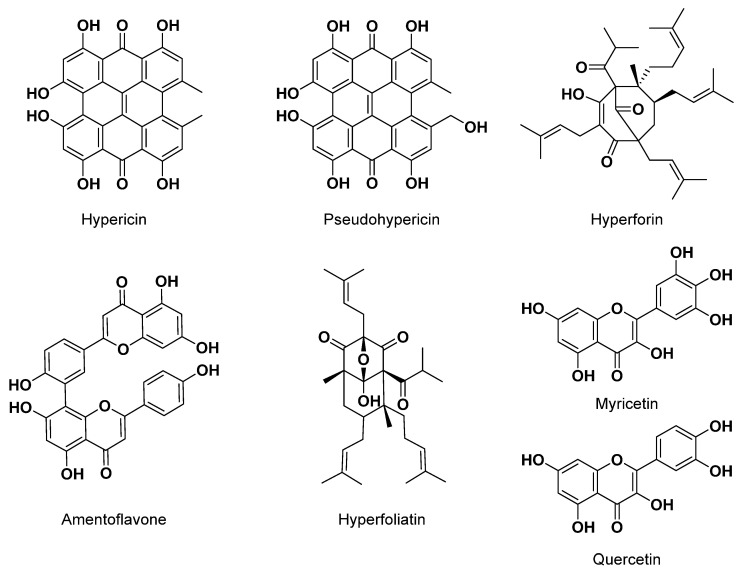
Selected secondary metabolites isolated from *Hypericum* species.

In another study on pure compounds isolated from *Hypericum perforatum* extracts, the in vitro pharmacologies of hypericin, pseudohypericin, and hyperforin, and several flavonoids were characterized at 42 biogenic amine receptors and transporters [162]. Amentoflavone demonstrated significant inhibitions of serotonin 5-HT1D, 5-HT2C, dopamine D3, and delta-opioid receptors, while hypericin displayed potent activities on dopamine D3 and D4, as well as adrenergic receptors. Notably, only the dopamine D1 receptor interacted with hyperforin [162]. The traditional use of the *Hypericum* species is not limited to depression, but is also used to treat wounds and burns, diarrhea, pain, and fever [163]. In fact, in addition to its traditional use in Algeria for treating depression and migraines, *Hypericum perforatum* is also reported to be used as an ointment, balsam, tincture to treat inflammation, antimicrobial agent, and wound healing agent [164]. Other *Hypericum* species found in Algeria have also been studied. For example, do Rego et al. found hyperfoliatin to be the metabolite responsible for the antidepressant effect of Algerian *Hypericum perfoliatum*, with the mechanism of action being associated with monoamine uptake inhibition [42]. From *Hypericum afrum*, another commonly used medicinal plant in Algeria, a series of flavonols, including quercetin and myricetin, were found to have neuroprotection properties and were identified as potent MAOs inhibitors [43]. Figure 6 shows a series of *Hypericum* metabolites with CNS activity.

Two intriguing herbaceous and aromatic genera commonly encountered in Algeria are *Salvia* spp and *Melissa* spp. These two genera belong to the Lamiaceae family and are primarily distributed in subtropical and tropical regions across the globe [165]. These two genera are renowned for their utilization as spices and their ethnopharmacological medicinal applications, with *Salvia officinalis* (Sage) and *Melissa officinalis* (Lemon Balm) being two prominent examples [166,167].

*Salvia* species are well-known for their abundance of essential oils and phenolic compounds, which encompass flavonoids and terpenes, particularly diterpenes. These compounds exhibit intriguing biological activities, notably an outstanding antibacterial and antioxidant potential [168,169,170]. Algeria hosts approximately 23 species of *Salvia*, with five of them being endemic [171]. In Algeria, *Salvia officinalis* is extensively employed in the northern regions for its traditional use in treating stress and insomnia, acting as a tranquilizer, and serving as a nervous tonic [34,72,172]. Recently, the neuroprotective effect of Algerian *Salvia officinalis* was investigated [173]. An aqueous extract of *Salvia officinalis* was administrated on rats exposed to an aluminum-induced neurodegeneration model. The study concluded that the extract of *Salvia officinalis* effectively reduced oxidative stress and improved biochemical parameters in the animals [173]. Other attractive Algerian *Salvia* species include *Salvia microphylla*, traditionally employed in Algeria for addressing memory loss and rheumatism [174].

On the other hand, *Melissa officinalis*, a Mediterranean medicinal plant, has been utilized for centuries to address CNS disorders, encompassing conditions such as depression, dementia, and various other ailments [175]. *Melissa officinalis* has been a valuable component of Algerian folk medicine, employed to address various neurological conditions, such as headaches, insomnia, migraines, nervousness, and depression [34,36]. Previous studies have shown that extracts of both *Melissa officinalis* and *Salvia elegans* exhibited a significant binding affinity to nicotinic and muscarinic acetylcholine receptors [176]. *Melissa officinalis* has also demonstrated potent neuroprotective effects [177].

**Figure 7:**
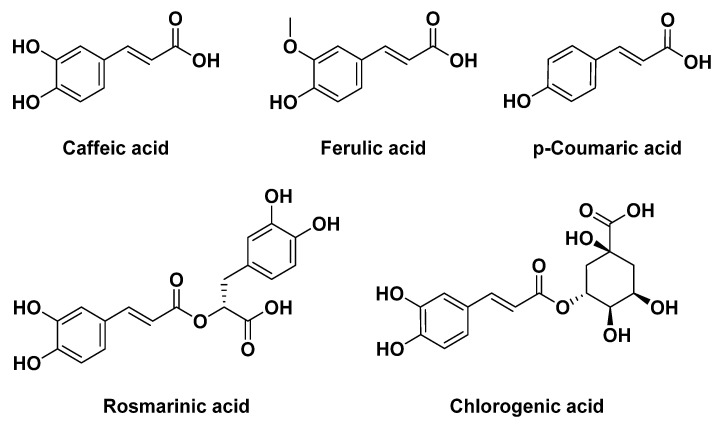
Bioactive phenolic acids from *Melissa officinalis.*

The chemical composition of *Melissa officinalis* includes flavonoids, phenolic compounds, and terpenoids [166]. Notably, essential oils and phenolic acids, such as caffeic, ferulic, chlorogenic, rosmarinic, and *p*-coumaric acids, are believed to be the primary bioactive constituents, acting synergistically to impart its medicinal properties (Figure 7).

*Thymus vulgaris* (common thyme) and *Rosmarinus officinalis* (rosemary) are two aromatic herbs belonging to the Lamiaceae family, commonly found in Algeria [34,72]. They are widely cultivated and can also be found growing wild in various regions of the country. Both *Thymus vulgaris* and *Rosmarinus officinalis* have a rich history of traditional use for culinary and medicinal purposes [31,34].

**Figure 8:**
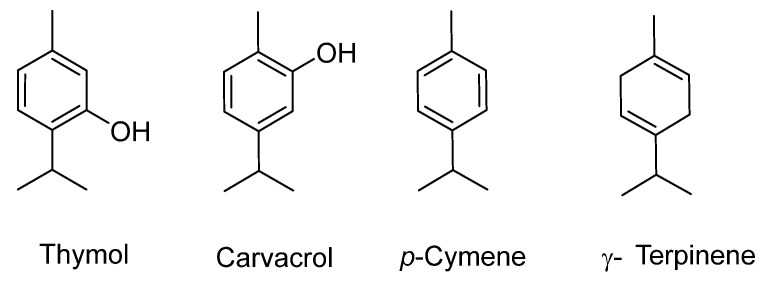
Major components of *Thymus numidicus* Poir. and *Thymus fontanesii* Boiss. & Reut. essential oils from Algeria [186,187].

*Thymus vulgaris* contains several bioactive compounds, notably thymol and carvacrol (see Figure 8) [31]. These two compounds are known for their potent antioxidant and anti-inflammatory properties. These properties have been linked to potential neuroprotective effects by reducing oxidative stress and inflammation, both of which are implicated in neurodegenerative diseases [178,179].

Thymol, at doses of 15 and 30 mg/kg, has been shown to increase the levels of central neurotransmitters and inhibit the expression of pro-inflammatory cytokines in a chronic unpredictable mild stress (CUMS) mouse model [180]. In a study conducted by Azizi et al. [181], thymol at doses of 0.5–2 mg/kg was shown to inhibit cognitive impairments caused by increased Aβ levels or cholinergic hypofunction in Aβ (25–35) or scopolamine-treated rats. This effect was attributed to thymol’s antioxidant, anti-inflammatory, and anticholinesterase properties. Lee et al. [182] demonstrated that thymol at concentrations of 0.39–25 mg/mL may inhibit H_2_O_2_-induced oxidative stress in PC-12 cells. Thymol at concentrations of 100 and 1000 mg/mL also inhibited both acetylcholinesterase (AChE) and butyrylcholinesterase (BChE) in a dose-dependent manner. Moreover, it has been reported that thymol at concentrations of 10–100 ppm, in combination with gamma-terpinene or para-cymene, attenuated cholinergic dysfunction, which is manifested in a plethora of neurodegenerative and psychiatric disorders, such as Alzheimer’s, Parkinson’s, and Huntington’s diseases, by enhancing the synaptic levels of acetylcholine (ACh) and the responsiveness of the nicotinic acetylcholine receptor (nAChR) in a *Caenorhabditis elegans* model [183]. According to the data presented by Zotti et al. [184], carvacrol is a brain-active molecule that influences neuronal activity through the modulation of neurotransmitters. Carvacrol was screened for pharmacological effects on the central nervous system and was found to present anxiolytic activity when administered orally to mice. Antidepressant effects were also observed in mice after carvacrol administration at doses of 12.5 to 50 mg/kg [184]. According to the researchers, the observed result was most likely due to an increase in dopamine levels. In a study conducted by Peters et al. [185], carvacrol was administered to mice after traumatic brain injury (TBI) and its effect on their functional recovery was followed for several weeks. The results showed that neurological recovery after TBI was significantly enhanced by the application of carvacrol. The authors found that neurological recovery after TBI was significantly enhanced by combining carvacrol with TRPC1 elimination [185].

Beyond its culinary appeal, rosemary has been treasured for its potential health benefits. It contains several bioactive compounds, including rosmarinic and carnosic acids, which possess antioxidant and anti-inflammatory properties [188]. These compounds have attracted interest in the field of natural medicine, and some studies have explored their potential neuroprotective effects on neurodegenerative diseases [188]. Rosemary’s aromatic properties have made it a popular choice for use in aromatherapy. Its essential oil is often used for relaxation, stress relief, and mental clarity [189]. In Algerian traditional medicine, rosemary has been used to support digestion, alleviate headaches, and promote overall well-being [36].

Rosmarinic acid, a polyphenolic compound found in high concentrations in rosemary, has been shown to have anti-epileptic activity by increasing the latency and decreasing the percentage of seizure incidents, reducing the levels of free radicals and DNA damage in a kindling CF-1 male mouse model of epilepsy induced by PTZ (rosmarinic acid at 1, 2, or 4 mg/kg b.w., i.p.) [190]. The administration of rosmarinic acid for 7 days at 5 and 10 mg/kg b.w./day led to the downregulation of mitogen-activated protein kinase phosphatase-1, the upregulation of BDNF, and the modulation of dopamine and corticosterone synthesis in a model of depression in mice with bupropion as a positive control [191]. In addition, rosmarinic acid has been shown to exhibit anti-tauopathy activity [192].

*Lavandula*, commonly known as lavender, is a fragrant flowering plant that belongs to the Lamiaceae family. It is native to countries bordering the Mediterranean Sea and is widely cultivated. *Lavandula angustifolia* (*Lavandula officinalis*), also known as true lavender, is the predominant species widely cultivated for its essential oil [193]. The plant’s essential oil finds extensive application in various industries, including perfumery, cosmetics, and aromatherapy, among others [193,194]. In Algeria, two Lavandula species, *Lavandula angustifolia* and *Lavandula stoechas*, hold a prominent position in traditional medicine [34,36]. Both of these plants are harnessed for their antispasmodic and wound disinfectant properties, making them valuable for addressing skin-related issues [72]. Additionally, these plants have been documented for their sedative and antidepressant attributes [72].

**Figure 9:**
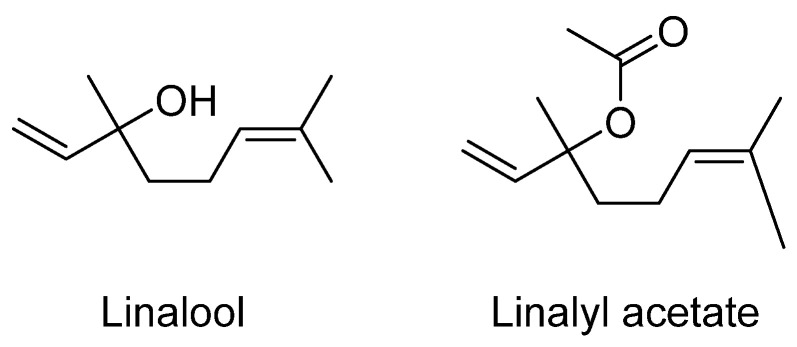
Some principle constituents of *Lavandula officinalis* essential oil grown in northeastern Algeria [197].

The local anesthetic effect of lavender oil and its major constituents, linalool and linalyl acetate (see Figure 9), has been reported in both in vivo and in vitro studies. An investigation of the effects of inhaled linalool on anxiety, aggressiveness, and social interaction in mice showed anxiolytic properties in the light/dark test, increased social interaction, and decreased aggressive behavior [195]. The anxiolytic effects of linalool odor were examined using the light/dark box test and the elevated plus maze (EPM). It was found that linalool odor has an anxiolytic effect without motor impairment in mice. Moreover, the effect was antagonized by flumazenil, indicating that the linalool odor-induced anxiolytic effect was mediated by γ-aminobutyric acid GABAergic transmission via benzodiazepine (BDZ)-responsive GABA_A_ receptors [196]. These results provide information about the potential central neuronal mechanisms underlying the odor-induced anxiolytic effects and lay the foundation for exploring the clinical application of linalool odor in anxiety treatments [196].

**Figure 10:**
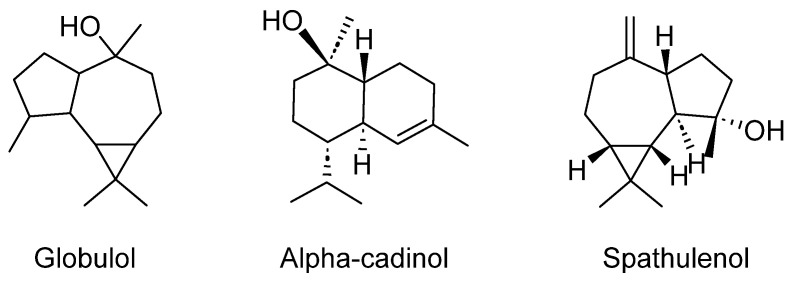
Main components of the essential oil of *Stachys circinnata* l’Her. From Algeria [201].

Species of the *Stachys genus,* a member of the Lamiaceae family, are recognized for their medicinal properties and are particularly valued for their distinctive aroma and flavor [93]. In Algeria, this genus is represented by 14 species, including four that are endemic to the region [171]. The main secondary metabolites identified from species in this genus encompass flavonoids, iridoids, fatty acids, phenolic acids, and diterpenoids [93]. Extracts and essential oils derived from *Stachys* species have exhibited promising anti-Alzheimer’s disease effects, primarily attributed to their ability to inhibit cholinesterase enzymes [198,199]. Figure 10 illustrates the key components of the essential oil extracted from Stachys circinnata l’Her. sourced from Algeria.

The effects of a hydroalcoholic extract and essential oil of *Stachys lavandulifolia* Vahl, a species used as an anxiolytic and sedative in Iranian folk medicine, were investigated using the elevated plus-maze (EPM) model of anxiety [200]. The *Stachys lavandulifolia* extract or its essential oil was administered intraperitoneally to male TO mice at various doses, 30 min before the behavioral evaluation. The results suggest that the extract of *Stachys lavandulifolia* possesses anxiolytic effects with relatively lower sedative activity compared to diazepam [200].

*Peganum harmala* L. (Nitrariaceae), commonly known as Harmal, is a perennial herbaceous plant thriving in arid and semiarid regions across various parts of the world, including North Africa, the Eastern Mediterranean, the Middle East, India, and other regions [202,203]. This plant has been used in traditional medicine for decades. Various parts of *Peganum Harmala*, including its seeds, bark, and root, are highly regarded for treating a wide range of human ailments [202,203]. *Peganum harmala* exhibits a diverse array of medicinal properties encompassing cardiovascular, antidiabetic, antimicrobial, insecticidal, antineoplastic, antiproliferative, gastrointestinal, and neurologic effects [202,203].

**Figure 11:**
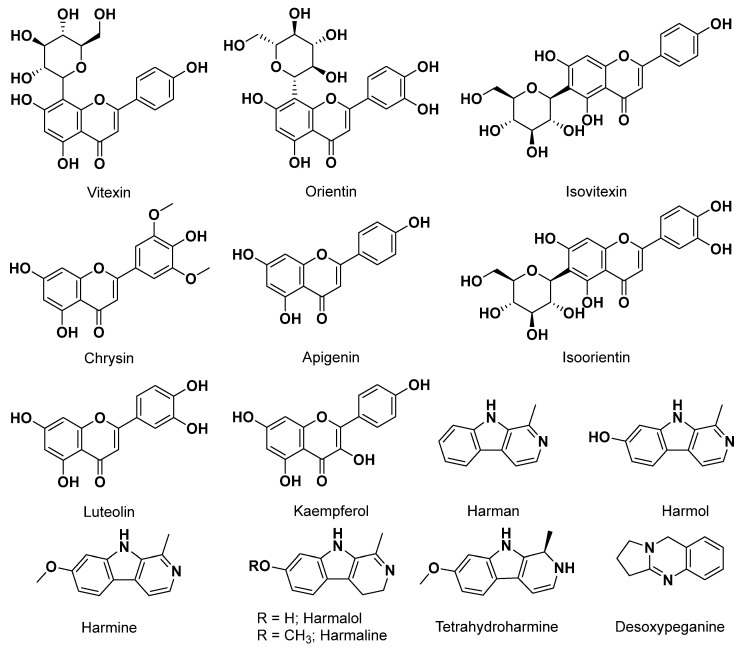
Selected secondary metabolites isolated from *Passiflora* and *Peganum harmala* species.

Algerian folk medicine, in particular, has harnessed the potential of *Peganum harmala* to address nervous system disorders and manage various psychiatric conditions, including nervosity, anxiety, and severe pain relief [34,72,202]. *Peganum harmala* holds a significant place in the fumigation rituals practiced across various cultures, including Algeria, which is believed to ward off the evil eye and bring good luck [202]. These rituals typically involve the burning of its seeds [72]. This tradition extends beyond Algeria and is observed in several other regions as well [204]. The smoke produced from the burning of these seeds is believed to have disinfectant properties [205]. Its composition includes alkaloids, essential oils, fatty acids, flavonoids, and anthraquinones [206]. Among these constituents, beta-carboline alkaloids, like harmalol, harmaline, and harmine, stand out as the most significant (Figure 11). These compounds can stimulate the central nervous system either by inhibiting amine neurotransmitter metabolism or through a direct interaction with specific receptors [207,208].

It has been proven that *Peganum harmala*-derived beta-carboline alkaloids interact with a plethora of targets in the CNS, including GABA (gamma-aminobutyric acid), NMDA (N-methyl-D-aspartate), glutamate, dopaminergic, serotonergic, and imidazoline receptors, as well as several enzymes, such as AChE, monoamine oxidase, and DyRK1A (dual-specificity tyrosine-phosphorylation-regulated kinase 1A), along with others, inducing many pharmacological effects [156,209]. Due to the fact that beta-carbolines are strong inhibitors of monoamine oxidase, they are potential agents to treat some neurological and psychological conditions [207]. As examples, Harmane, a beta carboline alkaloid isolated from *Peganum harmala*, induced amnesia through a mechanism of interaction with the dopaminergic system [210]. In a recent study, the extract of *Peganum harmala* was found to be able to enhance glucagon-like peptide 1 (GLP-1) and help to restore insulin signaling avoiding the progression of the AD [211].

*Passiflora* genus (Passifloraceae) includes perennial plants found largely endemically on the America’s (North, Central, and South Americas) [212]. They are mostly distributed in the warm, temperate, and tropical regions of the world. The *Passiflora* genus is another example of using plants for the treatment of insomnia, anxiety, and various other disorders of the CNS since antiquity; in fact, several *Passiflora* species have been documented for their use in the North/Central America Mayan empire [213]. *Passiflora* genus have been propagated and can been found in the tropical and subtropical areas in the world, and indeed in Algeria these species are usually planted in gardens and pergolas, but some are reported to be used in Algerian traditional medicine [72]. For example, *Passiflora edulis*, commonly known as passion fruit, has been used in Algerian folk medicine in the region of Skikda in northeast Algeria, is use being reported to treat hypertension and asthma, as well as a tranquilizer [214]. *Passiflora edulis* is a well-known crop cultivated around the world and highlighted for its nutritional and health benefits [215]. Another notable example is *Passiflora incarnata*, a medicinal plant used in northeast Algeria due to its effects on anxiety as a tranquilizer [72]. The potential effects of this species for treating some diseases, including opiate withdrawal, insomnia, attention-deficit disorder, and epilepsy, have also been widely reported [216,217,218,219,220]. Those two plants, and in general all *Passiflora* species, are a good source of flavonoids, particularly rich in C-glycoside flavones, such as vitexin, isovitexin, orientin, and isoorientin; flavones, such as apigenin, chrysin, and luteolin; and flavonols, including kaempferol and quercetin [221]. This species of β-carbolines alkaloids also includes harman, harmol, harmine, harmalol, and harmaline [222,223]. The overall effect of these plants has been ruled as being synergistic, however some of the compounds mentioned above have been studied, particularly to treat CNS disorders [223,224]. Figure 11 shows a series of bioactive compounds isolated from the *Passiflora* species.

With the traditional knowledge of *Passiflora incarnata’s* historical use in treating cannabis or cannabis product addiction, Dhawan et al. [225] conducted a study to explore the effect of the benzoflavone moiety from the *Passiflora incarnata* Linneaus plant on combatting cannabis addiction. When benzoflavone was administered at dosages of 10 and 20 mg/kg twice daily in combination with cannabinoids, specifically delta-9-tetrahydrocannabinol (Δ^9^-THC) at 10 mg/kg twice daily, a noteworthy outcome was observed. This combination resulted in a significant reduction in the tolerance to and dependence on cannabinoids. The benzoflavone component extracted from *Passiflora incarnata*, when administered simultaneously with Δ^9^-THC, effectively thwarted the development of a dependence on cannabinoids in the studied mice. Notably, even a single acute administration of the benzoflavone compound at a dose of 20 mg/kg via the oral route significantly inhibited the manifestation of withdrawal effects on Δ^9^-THC-dependent mice [225].

In diverse animal models designed to assess the anti-anxiety effects of *Passiflora incarnata*, the extracts consistently demonstrated pronounced anti-anxiety effects at the test doses employed [226]. This activity can be attributed to the presence of various secondary metabolites in the plant, with chrysin being a key compound responsible for its anxiolytic effects. Chrysin exerts its influence by modulating γ-aminobutyric acid GABA_A_ receptors. [226]. Both the extract and chrysin have been shown to display substantial anxiolytic activity in rodent models, as demonstrated through the elevated plus-maze (EPM) model of anxiety. The hypothesis suggests that chrysin mitigates anxiety by engaging with the GABA_A_ receptor in laboratory rats, as substantiated by assessments involving the elevated plus-maze, corticosterone levels, and catecholamine assays [226].

**Figure 12:**
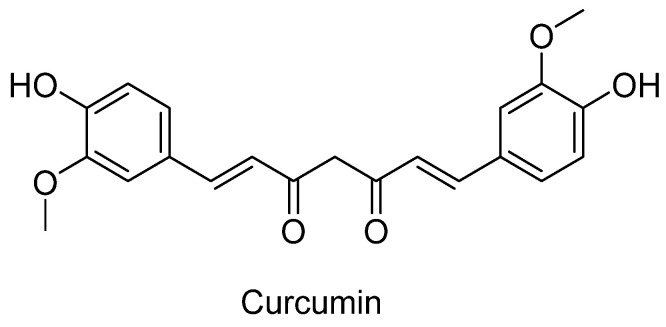
Curcumin, the main active molecule of *Curcuma longa* L.

*Curcuma longa* L., a plant belonging to the Zingiberaceae family, is commonly known as turmeric and is a popular spice in Algerian cuisine [227]. The local knowledge of turmeric in Algeria is primarily linked to its traditional use in Ayurvedic herbal medicine [227]. Its vibrant yellow color and unique flavor make it a favorite in many dishes. Beyond its culinary uses, turmeric has a long history of medicinal applications, dating back thousands of years, especially in traditional Indian and Chinese medicines [228]. The main active compound in turmeric is curcumin (Figure 12), which has garnered significant attention for its potential health benefits, including anti-inflammatory, antioxidant, and even anticancer effects [228].

Curcumin has been demonstrated to exert neuroprotective effects on a wide range of neurological disorders, including stroke, spinal cord injury, traumatic brain injury, AD, PD, multiple sclerosis, and epilepsy [229,230]. These neuroprotective properties of curcumin stem from its multifaceted attributes, including its role as an antioxidant, anti-inflammatory agent, anti-amyloidogenic compound, antidepressant, antidiabetic substance, and antiaging factor [231].

One of the most promising applications of curcumin in neurodegenerative disease therapy is its anti-amyloid property [232]. This property positions it as a highly promising compound for the treatment of various brain diseases characterized by amyloid accumulation. A recent review by Tang and Taghiglou [233] comprehensively summarized the latest developments in curcumin research related to AD. The review delved into the various mechanisms of action of curcumin in AD, encompassing its role in inhibiting Aβ and tau proteins, its capacity to bind copper, lower cholesterol levels, serve as an anti-inflammatory agent, modulate microglia, inhibit AChE, and function as an antioxidant. Furthermore, the review addressed topics concerning the bioavailability of curcumin and the current challenges encountered in curcumin therapy for AD patients [233].

Traditional medicine often incorporates at least one component or product derived from trees, such as fruits, flowers, bark, roots, seeds, resin, or oil [234,235].

*Populus nigra* L., a member of the Salicaceae family, is a cottonwood poplar species indigenous to North Africa, southern and central Europe, southwest and central Asia, and various other regions [236]. This tree thrives in numerous areas across Algeria, with its primary distribution occurring in the northern mountainous regions [237].

The therapeutic attributes of *Populus nigra* have been recognized since ancient times. This species is notable for its composition of phenolic glucosides, including salicin, poplin, and salicoside, as well as fatty acids, flavonoids, like flavones and flavanones, tannins, salicylates, and an essential oil containing sesquiterpenols, terpenes, and alkanes [5]. Notably, the buds of this tree contain the glucoside populoside benzoylosalicoside and are enriched with salicates, phenolic acids, and flavonoids [238].

*Populus nigra* buds are noted for their antiseptic properties and can also serve as a remedy for gout and hyperuricemia [238]. Furthermore, they have shown potential as a protective agent against neurodegenerative disorders [41]. Debbache-Benaida et al. conducted a study to investigate the effects of *Populus nigra* flower bud extract on brain modifications in mice subjected to aluminum-induced neurotoxicity. The study involved a battery of tests designed to assess various aspects of learning and memory functions, with the aim of evaluating the impact of the plant extract on AlCl_3_-induced neurotoxicity in mice [41]. Furthermore, the study examined the hypouricemic properties of the extract by measuring uric acid levels and liver xanthine oxidoreductase activity in both normal and hyperuricemic mice. The results demonstrated that the co-administration of *Populus nigra* extract (at a dose of 200 mg/kg) with AlCl_3_ (at a dose of 100 mg/kg/day), along with D-galactose (at a dose of 200 mg/kg/day) over a four-week period effectively counteracted the detrimental effects of AlCl_3_ by restoring all tested parameters. *Populus nigra* flower bud extract was proposed as a potential dietary supplement capable of mitigating the toxicity caused by AlCl_3_ and regulating serum uric acid levels [41,109].

**Figure 13:**
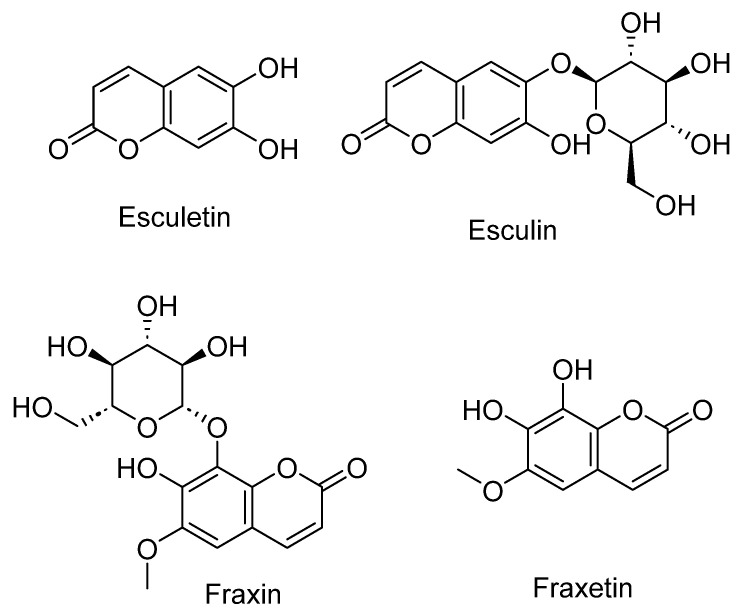
Selected hydroxycoumarins isolated from *Fraxinus* species.

*Fraxinus* (Oleaceae) is a genus of trees commonly referred to as ash trees, distributed across diverse regions of the world, with a presence in Algeria [5]. Algeria is home to the species *Fraxinus angustifolia,* which has a rich history of use in traditional medicine. *Fraxinus angustifolia* Vahl. has been used in traditional medicine for the relief of articular pain; prevention of gout; anti-inflammatory, antioxidant, diuretic, and digestive effects; and treatment of minor urinary infections [239]. It has also been suggested to have neuroprotective properties [240]. A range of bioactive phytochemicals, including secoiridoids, phenylethanoids, lignans, flavonoids, and coumarins, have been isolated from the Fraxinus plant [241]. Secondary metabolites, such as esculetin, esculin, fraxin, and fraxetin (Figure 13), and extracts from this plant have been found to present a range of biological activities, such as antioxidant, anti-inflammatory, antimicrobial, anticancer, hepatoprotective, antiallergic, skin regenerating, and diuretic [242,243]. Esculetin (coumarin) has been widely used in Chinese herbal medicine due to its pharmacological properties, such as antioxidant, anticancer, antibacterial, and anti-inflammatory [243].

In a recent study by Azib et al., the neuroprotective potential of *Fraxinus angustifolia* Vahl. bark extract was investigated both in vitro and in vivo. The study aimed to assess its efficacy in mitigating Aβ-aggregation and alleviating aluminum-induced neurotoxicity in mice [244]. The neuroprotective properties of the extract were assessed in various experimental setups. Initially, its efficacy against Aβ25-35 aggregation was determined by direct incubation with Aβ25-35, and the kinetics of aggregation were monitored using a spectrophotometer at 200 nm. Subsequently, the extract was subjected to an evaluation against Aβ25-35-induced cytotoxicity in PC12 cells, with the cell viability assessed through an MTT test [244]. Furthermore, *Fraxinus angustifolia* Vahl. Bark extract was tested across a range of concentrations (0.01–0.5 mg/mL) to gauge its impact on aluminum-activated lipid peroxidation in the synaptosomal membranes of mice. In vivo experiments were conducted on N.M.R.I. male mice to investigate its potential in countering aluminum-induced neurotoxicity. The results demonstrated that the extract exhibited a pronounced anti-aggregative effect against Aβ25-35 and displayed a dose-dependent protective effect on PC12 cells. Additionally, the extract showcased a significant inhibition of lipid peroxidation and effectively mitigated the detrimental effects of aluminum. This was evident through notable enhancements in locomotor activity, reduced anxiety, improved memory, and a decrease in histological alterations [244].

*Juniperus* (Cupressaceae) is a genus encompassing both trees and shrubs, widely recognized as junipers, with a longstanding history of traditional medicinal usage in Algeria. In Algerian flora, there are five notable species of junipers [1] Among these, two prominent species stand out: *Juniperus oxycedrus* L. and *Juniperus phoenicea* L.

*Juniperus oxycedrus* L., commonly referred to as Cade, is a species primarily distributed throughout the Mediterranean region [78]. In Algerian traditional medicine, it is revered for its diuretic, stimulative, and stomachic tonic properties [57]. Furthermore, it is highly valued for its effectiveness as a pulmonary and depurative disinfectant, in addition to its various other applications [5]. *Juniperus phoenicea* L. is another species indigenous to the Mediterranean region, including Algeria. In a study conducted to assess traditional knowledge and the utilization of medicinal plants by Algerian traditional healers, this species exhibited an exceptional fidelity value of 100% [36].

*Juniperus* species have garnered significant attention due to the diverse bioactivities associated with the extracts and secondary metabolites derived from these plants [245]. These bioactivities encompass a broad range of beneficial effects, including antimicrobial, antioxidant, antidiabetic, anti-inflammatory, anticonvulsant, analgesic, and cytotoxic activities [245]. Furthermore, the research findings highlight their potential as neuroprotective agents, suggesting promising attributes in supporting and safeguarding the health of the nervous system [66,246].

**Figure 14:**
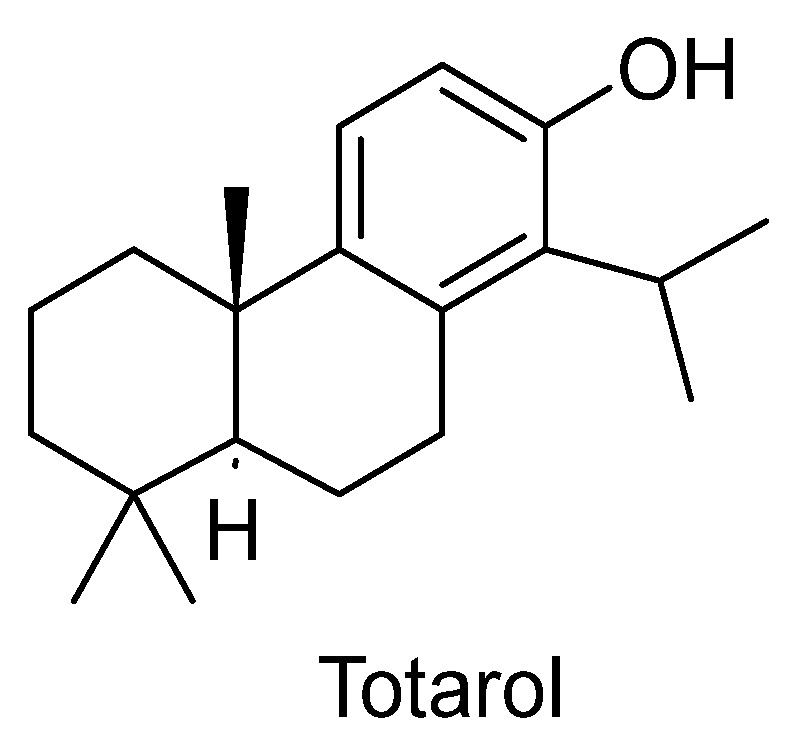
Totarol, a molecule found in the *Juniperus* species.

A study conducted by Gao et al. [247] revealed the vascular protective effects of totarol (Figure 14), a tricyclic phenolic diterpene abundantly present in various *Juniperus* species [248]. The study demonstrated that totarol activated the protein kinase B/heme oxygenase-1 (PKB/HO-1) pathway, leading to increased levels of superoxide dismutase (SOD) and the antioxidant glutathione (GSH). This, in turn, suppressed ischemia-induced brain injury. To simulate the conditions of patients with acute strokes, rats were administered totarol post-ischemia at doses of 1 and 10 µg/kg. The results revealed a significant reduction in the infarct volume compared to the untreated group. Furthermore, totarol treatment at both 1 and 10 µg/kg significantly enhanced the ischemia-induced response [247].

### 4.7. Clinical Trials and Therapeutic Applications

Clinical trials are research studies involving human participants, focusing on evaluating the efficacy and safety of new treatments, such as novel drugs or diets.

Medicinal plants continue to be a significant source of new drugs, drug leads, and chemical entities [249]. The research on the drug discovered in plants has led to the development of numerous bioactive agents, including those with antidiabetic, anticancer, anti-infectious, anti-withdrawal, antianxiety, and antidepression properties, among others [250]. However, despite the burgeoning body of in vitro and in vivo investigations into medicinal plant extracts and their active constituents, a dearth of comprehensive assessments pertaining to the safety and efficacy of herbal medicines persists, largely attributable to an array of challenges confronting researchers in the domain of herbal drug discovery. These challenges include difficulties in accessing expensive screening methods, technological limitations that hinder the full analysis of phytochemical complexity, and the significant time required to conduct a comprehensive phytochemical study [251,252]. In contrast, pharmaceutical companies have achieved remarkable success in drug discovery research, demonstrated by their rapid development of potential vaccines for the COVID-19 pandemic in 2020 [253,254].

In recent years, there has been a notable increase in studies related to clinical trials on medicinal plants and their main active components. In 2005, the World Health Organization (WHO) issued operational guidelines outlining the regulatory requirements to support clinical trials of herbal products [84].

In Algeria, conducting clinical trials on pharmaceutical products, herbal preparations, or natural bioactive compounds is severely restricted, and the process is subject to complex laws [255]. Numerous species of *Hypericum* have undergone thorough investigations to explore their pharmacological and phytochemical properties, particularly concerning their clinical efficacy in treating mild to moderate depression [256]. St. John’s wort (*Hypericum perforatum*) is a herbal product known for its clinically significant effects [257]. In a single-blind clinical study conducted by Dimpfel et al., the effects on the CNS of two commercially available extracts of St. John’s wort (Texx 300 and Jarsin 300) were compared with those of a placebo in a group of healthy young volunteers (*n* = 35) [258]. Both extracts showed a decrease in the cognitive potential P300, suggesting an enhanced mental performance. The observed neurophysiological changes were consistent with the proposed clinical efficacy. Moreover, quantitative EEG allowed for a discrimination between St. John’s wort extracts with respect to the time of effect and profile changes on the neuronal communication structure [258].

*Melissa officinalis*, also known as lemon balm, is traditionally used as a mild sedative, for memory enhancement, and for treating anxiety, depression, and insomnia [34,259]. It has been reported to have many biological activities, such as antioxidant, antimicrobial, antitumor, antiviral, antiallergic, anti-inflammatory, and flatulence-inhibiting effects [175]. The beneficial properties of *Melissa officinalis* can be related to bioactive compounds, such as terpenoids, alcohols, rosmarinic acid, and phenolic antioxidants, which are present in the plant [175]. In a clinical study conducted by Safari et al., *Melissa officinalis* extract demonstrated a significant impact on reducing depression and anxiety in patients with type 2 diabetes [260]. Another study evaluated samples of *Melissa officinalis* for their potential to inhibit human AChE and bind to cholinergic receptors [261]. Subsequently, the cognitive and mood effects of single doses of the most cholinergic active dried leaf extract were assessed in a randomized, placebo-controlled, double-blind, balanced crossover study. Twenty healthy, young participants received single doses of 600, 1000, and 1600 mg of encapsulated dried leaf, or a matching placebo, at 7-day intervals. Cognitive performance and mood were evaluated before the dose and at 1, 3, and 6 h post-dose using the Cognitive Drug Research computerized assessment battery and Bond–Lader visual analog scales, respectively. The most prominent cognitive and mood effects consistently observed at all post-dose time points were the enhancement of memory performance and an increase in feelings of calmness, particularly noticeable with the highest dose of 1600 mg. These findings suggest that doses of *Melissa officinalis* equal to or exceeding the maximum dosage utilized in the study have the potential to improve cognitive function and mood. As a result, they may hold promise as a valuable adjunct in the treatment of AD.

Furthermore, these results underscore the significance of considering that different preparations derived from the same plant may exhibit diverse characteristics based on the specific sample preparation methods employed.

In an 8-week randomized, double-blind clinical trial designed to assess the effectiveness of *Melissa officinalis* and *Lavandula angustifolia* in comparison to fluoxetine for treating mild to moderate depression, 45 adult outpatients meeting the criteria outlined in the Diagnostic and Statistical Manual of Mental Disorders, 5th edition (DSM-5), for major depression were randomly divided into three groups. These groups were assigned to daily doses of either *M. officinalis* (2 g), *L. angustifolia* (2 g), or fluoxetine (20 mg). Evaluations were conducted at weeks 0, 2, 4, and 8 using the Hamilton Rating Scale for Depression (HAM-D), which comprised 17 items [262]. The results indicate that both *Melissa officinalis* and *Lavandula angustifolia* have a similar effect to fluoxetine when treating mild to moderate depression (F = 0.131, df = 2.42, *p* = 0.877).

The selection of *Salvia officinalis* species for clinical trials was grounded in its well-established properties and supported by a substantial body of evidence derived from both in vitro and in vivo studies. These clinical trials have a primary objective of evaluating its potential beneficial impact on cognitive function, targeting both healthy individuals and patients coping with cognitive impairments, such as AD [263].

In a controlled, double-blind, crossover study, a total of 30 healthy participants visited the laboratory on three separate occasions, spaced 7 days apart. During each visit, they were administered a different treatment in a counterbalanced sequence, which included a placebo and two doses of dried sage leaf (300 and 600 mg). Mood assessments were conducted both before taking the treatment and at 1 and 4 h after ingestion. These mood assessments involved the completion of Bond–Lader mood scales and the State Trait Anxiety Inventory (STAI), both before and after engaging in a 20 min session of the Defined Intensity Stress Simulator (DISS) computerized multitasking battery [263]. In a correlated study, an extract derived from *Salvia officinalis* leaves demonstrated a dose-dependent inhibition of AChE and, to a more pronounced extent, butyrylcholinesterase in in vitro experiments. The lower dosage was observed to alleviate anxiety, while the higher dosage heightened feelings of “alertness”, “calmness”, and “contentedness”, as measured by the Bond–Lader mood scales. These findings provide further validation of previous research concerning the cholinesterase inhibitory characteristics of *Salvia officinalis* [263].

The well-known plant *Passiflora incarnata*, often used as a calming herb to treat anxiety and insomnia, was the subject of a clinical study conducted by Movafegh et al. [264]. In this study, the effectiveness of *Passiflora incarnata* in controlling anxiety in preoperative patients was investigated. The plant extract was consumed as tea and showed positive effects on the quality of sleep [264]. In the study, 60 patients were randomized into two groups to orally receive *Passiflora incarnata* (500 mg, Passipy™ IranDarouk) (*n* = 30) or placebo (*n* = 30) as premedication, 90 min before surgery. A numerical rating scale (NRS) was used for each patient to assess anxiety and sedation before and 10, 30, 60, and 90 min after premedication. The results revealed that the NRS anxiety scores were significantly lower in the Passiflora group compared to the control group (*p* < 0.001). Importantly, the study suggested that the administration of oral *Passiflora incarnata* as a premedication effectively reduced anxiety without inducing sedation in outpatient surgery [264].

### 4.8. Toxicological Evidence

The widespread availability of plants renders them easily accessible for various applications, with the majority of these plants generally regarded as safe. Nonetheless, it is crucial to acknowledge that certain plants can elicit adverse secondary effects, including harmful toxic reactions. In specific instances, the utilization of particular plants or specific plant components can even prove fatal. The toxic properties of these plants emanate from the presence of active compounds distributed throughout the entire plant or in one or more of its constituent parts, such as the leaves, fruits, or roots. These compounds are associated with specific organ toxicity in both humans and animals, leading to a spectrum of disorders that can range from mild to severe, and in extreme cases may culminate in fatality.

Various plants and their active constituents, as outlined in Table 1, have been linked to toxicity. For instance, wormwood (*Artemisia absinthium* L.) was banned in numerous countries during the early 20th century due to its neuropsychiatric toxicity. This toxic effect can be attributed to thujones, which are volatile monoterpene ketones. Thujones make up approximately 0.25–1.32% of the entire herb and 3–12% of the essential oil. The neurotoxic mechanism of thujones has undergone extensive investigation in animal studies, cultured neuronal cells, and studies involving expressed receptors [151]. Among thujones, α-thujone is roughly two-to-three-times more potent than β-thujone in modulating the GABA-gated chloride channel. It is worth noting that the neuronal effects of thujone were found to be fully reversible [151]. Thujone has been documented as toxic to the brain, liver, and kidney cells, and its ingestion in excessive amounts can lead to various adverse effects, including convulsions (muscle spasms), wheezing, restlessness, anxiety, insomnia, vomiting, vertigo, rapid heart rate, kidney damage, epileptic seizures, and even psychedelic effects [151].

**Figure 15:**
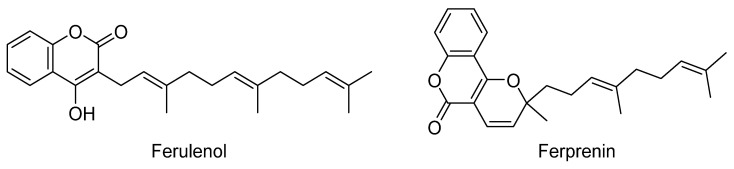
Toxic components present in *Ferula communis* L.

*Ferula communis* L. is a common Mediterranean plant of the Apiaceae family. It has traditionally been used to treat anxiety and hysteria [50,265]. The rhizomes of this plant, known as Al-kalakh in Arabic, are used locally as a traditional remedy for the treatment of skin infections [50,52]. In vitro and in vivo studies have shown that the plant’s crude extracts and isolated components possess various pharmacological properties, including antidiabetic, antimicrobial, antiproliferative, and cytotoxic activities [265]. The toxicity of *Ferula* has been well-documented in North Africa for an extended period of time [50]. Incidents of poisoning have been frequently reported in various animal species, particularly sheep, cattle, pigs, horses, and goats, often occurring after several days of grazing in the fields. Additionally, cases of *Ferula*-induced toxicity have been documented in humans [265]. Exposure to *Ferula communis* can lead to a hemorrhagic syndrome known as ferulosys [266]. Active compounds, notably the prenylated coumarin derivatives ferulenol and ferprenin (Figure 15), have been identified as inhibitors of the vitamin K recycling process in liver microsomes, specifically targeting the enzymatic system known as vitamin K epoxide reductase (VKORC1) [267]. The inhibition of VKORC1 by these 4-hydroxycoumarin derivatives limits the availability of KH2 for the carboxylation reaction, resulting in the partial carboxylation of vitamin K-dependent blood-clotting factors II, VII, IX, and X. The specific inhibition of VKORC1 leads to the cessation of clotting factors’ activation, ultimately culminating in a fatal hemorrhage [267].

A study conducted by Lahmar et al. [268] reported on *Ferula communis* intoxication in goats. They determined the acute LD50 of ferulenol in albino mice to be 2100 mg/kg bw after a single oral administration and 319 mg/kg bw after an intraperitoneal administration. Notably, ferulenol exhibited a higher LD50 compared to warfarin, indicating lower acute toxicity. Three days following the ferulenol administration, the dosed animals exhibited hypoprothrombinemia, accompanied by both internal and external hemorrhages resembling the symptoms described in ferulosis and experimental anti-vitamin K poisonings [268].

Merino sheep in a flock located on the southwestern slopes of New South Wales experienced intoxication, serving as yet another instance of neurological toxicity in animals resulting from the consumption of specific plant species while grazing in the fields [269]. After consuming *Stachys arvensis*, a plant belonging to the Lamiaceae family, these animals developed a neurological disorder, with numerous fatalities linked to the outbreak. The impacted sheep exhibited symptoms consistent with mild degenerative myelopathy and peripheral neuropathy. Additionally, deficiencies in vitamins A and E were identified within the affected flock. Clinical examinations and post-mortem analyses were conducted on the sheep displaying clinical signs. The research concluded that grazing on *Stachys arvensis* is at times linked to a neurological locomotor disorder in sheep [269].

*Peganum harmala* L. is a plant with a broad distribution across the Mediterranean region, including Algeria. It is commonly utilized in traditional medicine for its sedative properties and various other purposes, but its use comes with the inherent risk of overdose and poisoning for users [203]. The intoxication caused by *Peganum harmala* L. seeds has been widely reported in both animals and humans [270,271]. Ingesting high quantities of *Peganum harmala* can lead to various clinical manifestations of intoxication, including digestive disorders, bradycardia, neurological symptoms, such as euphoria, hallucinations, generalized tremors, and, in severe cases, it can lead to paralysis, central nervous system depression, dyspnea, and arterial hypotension [272]. The main alkaloid components found in *Peganum harmala* seeds consist of the beta-carboline alkaloids harmine, harmaline, and tetrahydroharmine [203]. These compounds are also detectable in other plants, like *Passiflora incarnata*, and are notably present in the psychoactive brew called Ayahuasca [273]. Ayahuasca is a hallucinogenic beverage with roots in indigenous Amazonian traditions, where it has been employed in religious ceremonies and therapeutic rituals [274]. The toxicokinetics and toxicodynamics of the psychoactive alkaloids harmine, harmaline, and tetrahydroharmine have undergone extensive examination. Comprehensive documentation exists on the psychological, physiological, and toxic effects resulting from their simultaneous consumption [275].

**Figure 16:**
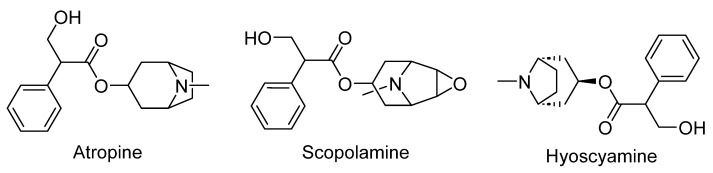
Toxic tropane alkaloids identified in *Datura stramonium* growing in Algeria [283,284].

*Datura stramonium* and *Atropa belladonna belong* to the Solanaceae family and have a rich historical background as hallucinogenic substances [276]. These plants have also played a significant role in traditional medicine and have been linked to practices involving sorcery, witchcraft, and magico-religious rituals. It is important to note that both of these plants contain the active hallucinogenic compounds atropine and scopolamine [277]. Tropane alkaloids are important natural products that are abundantly present in the Solanaceae family and include the anticholinergic drugs atropine, hyoscyamine, and scopolamine (Figure 16) [278].

Instances of Datura intoxication can arise from various causes, including medication overdoses, the misuse of edible plants, intentional abuse for hallucinogenic purposes, criminal activities, such as homicide or robbery, and accidental poisoning from contaminated food [279]. Reported symptoms of toxicity encompass dizziness, dry mouth, flushed skin, palpitations, nausea, drowsiness, tachycardia, blurred vision, mydriasis (dilated pupils), hyperthermia, disorientation, vomiting, agitation, delirium, urine retention, hypertension, and even coma [279]. The belladonna alkaloids (atropine, hyoscyamine, and scopolamine) present in the roots, leaves, and fruits of these plants pose a substantial risk, particularly to infants, children, and adolescents, and can result in an anticholinergic toxidrome [280]. Cases of anticholinergic poisoning stemming from contamination with belladonna alkaloids have been reported in various food items, such as commercially purchased Paraguay tea, hamburgers, and honey [281]. Additionally, there have been other documented instances of tropane alkaloid-related intoxications, including a significant epidemic in New York and the eastern United States, attributed to heroin contamination with scopolamine [282].

While essential oils have a wide range of applications, they should be used with caution due to their potential toxic effects, such as phototoxicity or photosensitivity. In some cases, high doses of certain essential oils can lead to hepatotoxicity, nephrotoxicity, and neurotoxicity [285]. For instance, previous studies recommend that oregano essential oil should never be applied topically to mucous membranes in concentrations higher than 1% due to its potential for skin irritation and even a burning sensation [286]. The main compounds in oregano essential oil, carvacrol and thymol (Figure 8), can be toxic to the liver, kidneys, and nervous system if consumed in excessive quantities. Furthermore, carvacrol and thymol have demonstrated in vivo mutagenicity and genotoxicity [287,288].

The essential oils derived from the *Juniperus* species exhibit a diverse range of pharmacological properties. Nevertheless, it is crucial to highlight that there have been numerous reported cases of intoxication associated with cade oil, obtained from the branches of *Juniperus oxycedrus* L. (Cupressaceae) [289,290,291]. Phenol, a constituent of cade oil, can exert adverse effects on various organ systems, including the central and peripheral nervous systems, cardiovascular system, liver and biliary systems, skin, and respiratory system [291].

In a study aimed at assessing the toxicity of constituents found in cade essential oil, *Juniperus oxycedrus* L., researchers identified eight structurally related compounds and an additional 16 previously known cade oil constituents that were toxic to adult American house dust mites (AHDMs) [292]. Among these compounds, methyl-eugenol (LD50, 5.82 µg/cm^2^) and guaiacol (LD50, 8.24 µg/cm^2^) demonstrated the highest toxicity against the mites. It is worth noting that the toxicity of these compounds, as well as benzyl benzoate, do not exhibit significant differences. Additionally, eugenol, *m*-cresol, and nerolidol (LD50, 12.52–19.52 µg/cm^2^) exhibited notably high toxicity levels compared to *N*,*N*-diethyl-3-methylbenzamide (DEET) (LD50, 37.67 µg/cm^2^). When cade oil was applied experimentally as sprays at concentrations of 30 or 40 mg/L, it resulted in 96% and 100% mortality rates among the mites, respectively. In contrast, the application of permethrin (cis:trans, 25:75) at a concentration of 2.5 g/L via spray treatment only yielded a 17% mortality rate [292].

## 5. Materials and Methods

Information regarding the traditional use of medicinal plants for the management of mental illnesses and central nervous system (CNS) disorders in Algeria was gathered through an extensive literature search. This encompassed sourcing data from books, e-books, encyclopedias detailing medicinal plant uses, as well as Master’s and PhD theses focused on the subject matter. Additionally, various scientific databases, including Scifinder^®^, Scopus, Scholars, PubMed, Medline, Web of Science, the World Health Organization (WHO), SFE (Societe Francaise d’Ethnopharmacologie), and Google Scholar, were meticulously scrutinized for historical and ethno-pharmacological information, biological and pharmacological actions.

The search parameters were confined to specific keywords, including “treatment of mental illnesses with medicinal plants”, “Algeria”, “North Africa”, “Algerian medicinal plants”, “pharmacopeia”, “neuropsychiatric disorders”, “in vitro study”, “in vivo study”, “clinical trials of medicinal plants”, “secondary metabolites and CNS interaction”, and “natural drug interactions and toxicity”. All identified papers underwent a thorough inspection for relevance, and additional sources were identified by examining reference lists.

## 6. Conclusions and Prospects

Algeria’s vast and diverse territory, enriched with abundant biodiversity and a variety of landscapes, stands as a testament to its unique geographical location and historical interactions with diverse civilizations and cultures. This historical tapestry has profoundly influenced the development of Algeria’s pharmacopeia, creating a rich repository of knowledge regarding the medicinal properties of countless herbs. The indigenous wisdom held by Algeria’s local population concerning medicinal plants is an invaluable treasure trove of insights.

Nonetheless, there exists an urgent imperative to systematically document the medicinal applications of Algerian plants. This urgency stems from the alarming rate of natural habitat degradation, driven by factors like overexploitation, wildfires, anthropogenic activities, and the fading expertise of traditional healers. Together, these factors contribute to the unfortunate erosion of precious traditional knowledge.

To address this pressing need, securing funding for dedicated research initiatives becomes paramount. These initiatives should focus on conducting comprehensive investigations into specific plant species native to Algeria. The primary objective of these studies would be to assess their potential effectiveness in treating brain disorders and neurodegenerative diseases, as well as to pinpoint the bioactive compounds responsible for their pharmacological effects.

Looking ahead, our primary focus should center on the meticulous selection of the most promising plants, particularly those demonstrating evident effects on the central nervous system yet lacking sufficient scientific scrutiny to date. By delving into thorough pharmacological examinations of these selected plants, we can unlock the secrets of their bioactive molecules and elucidate their mechanisms of action. This approach not only safeguards Algeria’s rich herbal heritage but also holds promise for discovering novel treatments for neurological ailments, ultimately benefiting both local communities and the broader scientific community.

## Figures and Tables

**Table 1:** Medicinal plants used in the management of mental illnesses in Algeria.

S/No	Plant Name	Family	Common Name	Local Algerian Name(s)	Use for Mental Disorders	Plant Part/s Used	Traditional Preparation and Administration Methods	Identified Active Constituent(s)	Other Relevant Phytoconstituents Identified in the Plant	Interaction/Toxicity	References
1	*Narcissus tazetta* L.	Amaryllidaceae	Narcisse à bouquet	Nardjes	Epilepsy, memorigenic, hysteria, AD.	Roots, flowers, bulbs.	Infusion.	Alkaloids (galanthamine, lycorine, homolycorine, tazettine, narciclasine).	Flavonoids, saponins, tannins, cardiac glycosides, essential oil, steroids, terpenoids, anthraquinones.		[45,46,47]
2	*Pistacia lentiscus* L.	Anacardiaceae	Pistachier lentisque	Dharw	Memory.	Leaves, fruits, resin, essential oil.	Decoction, infusion, fruits can be naturally eaten raw.	Tannins, essential oil (monoterpenes), triterpenes.	Polyphenols, phytosterols, flavonoids, triglycerides, tocopherols, carotenoids.	Toxic at higher doses.	[34,48,49,50]
3	*Coriandrum sativum* L.	Apiaceae	Coriandre	Kousbor	Epilepsy, nervous tension, tranquilizer, migraine.	Essential oil, leaves, seeds.	Decoction, infusion, can be naturally chewed.	Essential oil (coriandrol, pinenes, terpinenes, borneol, linalool, geraniol).	Aromatic acids, Isocoumarins, polyphenols.		[46,51]
4	*Ferula communis* L.	Apiaceae	La Férule	Fessoukh, Kelekh	Anxiety, anti-hysteria.	Gum resin (latex), roots, leaves, stems	Decoction infusion powder cataplasm fumigation.	Coumarins, sesquiterpene prenylated coumarins (ferulenol).	Daucane esters, phenylpropanoids, phenolic compounds.	Toxic (ferulenol, 4-hydroxycoumarin derivatives, ferprenin).	[5,50,52]
5	*Ferula assa-foetida* L.	Apiaceae	Férule persique	Hantit	Epilepsy, tranquilizer, stimulant to the brain and nerves.	Oleo-gum resin, aerial parts, seeds, roots, young shoots and leaves.	Decoction, powder.	Essential oil, sesquiterpene, coumarins (foetidin).	Disulfides, ferulic acid, valeric acid.		[53,54]
6	*Pimpinella anisum* L.	Apiaceae	L’anis vert	Habet h’lawa, Yansoune	Insomnia.	Essential oil, seeds.	Infusion.	Essential oil (anethol, methyl chavicol) furanocoumarins, flavonoids.	Sterols, proteins, fatty acid, terpenes.	May be toxic under certain conditions.	[55,56]
7	*Anacyclus pyrethrum* (L.) Lag.	Asteraceae	Pyrèthre d’Afrique	Agargarha, Kantass	Epilepsy, paralysis, seizures, depression, anxiety.	Roots, essential oil.	Decoction, chew, lozenge, powder.	Essential oil, anacycline, inulin.	Pellitorine.		[5,54]
8	*Artemisia absinthium* L.	Asteraceae	L’absinthe	Chadjrat meriem	Insomnia, slightly antidepressant.	Aerial parts, leaves.	Infusion, maceration, decoction.	Essential oil, terpenes, azulenes, thujone, sesquiterpene lactones (artabasine, absinthin).	Phenolic compounds, lignans.	Essential oil constituents are highly toxic (alpha-thujone and beta-thujone).	[34,54,57,58]
9	*Artemisia herba alba* Asso.	Asteraceae	L’armoise	Chih	AD, epilepsy, depression, neuroinflammation.	Aerial parts.	Infusion, decoction.	Essential oil, herbalbin, cis-chryanthenyl acetate, flavonoids (hispidulin and cirsilineol), monoterpenes, sesquiterpene lactones.	Coumarins, tannins.	Toxic at over dose.	[5,34,50,57]
10	*Carlina gummifera* (L.) Less. Syn. *Atractylis gummifera* L.	Asteraceae	Le chardon à glu	Addad	Epilepsy, seizure management, mania.	Capitulum, leaves, roots.	Decoction.	Polyphenols, tannins.	Diterpenoid glucosides.	Roots are highly toxic (mortal).	[36,50,59,60,61]
11	*Chamaemelum Nobile syn. Anthemis nobilis* L.	Asteraceae.	La Camomille romaine	Babounej	Anxiety.	Flowers, essential oil.	Infusion, decoction.	Essential oil (angelic acid esters, chamazulenes), sesquiterpene lactones (nobilin), flavonoids, coumarins.	Polyphenols.		[5,34,54]
12	*Matricaria chamomilla* L.	Asteraceae	La Camomille sauvage	Babounej	Migraines, insomnia.	Capitulum.	Infusion.	Essential oil (alpha-bisabolol, chamazulene), flavonoids, coumarins, tannins.	Polyphenols.		[36,62]
13	*Scolymus hispanicus* L.	Asteraceae	Chardon d’ Espagne	Zernich, Guernina	Different neurological conditions.	Roots, stems, leaves, flowers.	Infusion.	Flavonoids, tannins.	Phenolics.		[63,64,65,66]
14	*Silybum marianum* (L.) Gaertner	Asteraceae	Chardon marie	Chouk	Depression.	Seeds.	Dried seeds, decoction, tincture.	Silymarin, silybin A, silybin B, isosilybin A, isosilybin B, silychristin A, silydianin, taxifolin.	Polyphenols, essential oil, tannins.		[5,63]
15	*Berberis vulgaris* L.	Berberidaceae	L’épine-vinette	Oud ghriss	Sedative, morphine addiction.	Leaves, fruits, stem bark, roots.	Raw, decoction, infusion.	Berberine.	Alkaloids (berbamine, jateorrhizine, palmatine, oxycanthine).	Toxic in higher doses.	[54,67,68,69,70]
16	*Lepidium sativum* L.	Brassicaceae	Cresson alénois	Hab err-chad	Insomnia, memory.	Seeds.	Powder.	Tannins, vitamins, minerals.	Flavonoids, carbohydrates, phenolics, alkaloids, proteins, saponins, lipids.		[71,72,73]
17	*Commiphora myrrha* (Nees) Engl.	Burseraceae	Myrrh	El-morra	Memory impairment, tranquilizer, anxiety.	Gum resin, seeds.	Infusion, powder.	Essential oil, sesquiterpenes, furanosesquiterpenes, polysaccharides, tannins.	Proteins and long-chain aliphatic derivatives, steroids, sterols, terpenes.		[5,74]
18	*Boswellia sacra* Flueck.	Burseraceae	Oliban	Loubene	CNS disorders, AD, depression, mental fatigue, stress.	Gum resin, stems.	Powder, infusion, maceration, mastication, decoction, fumigation.	Boswellic acids.	Essential oil, phenols, terpenoids, uronic acids, steroids, tannins.		[5,54]
19	*Opuntia ficus-indica* (L.) Mill.	Cactaceae	Figuier de Barbarie	Tine chawki, El-hendi	Headache, dizziness.	Leaves.	Decoction.	Flavonoids, polyphenols.	Polysaccharides, sterols, omega-3 fatty acid.		[31,36,75]
20	*Humulus lupulus* L.	Cannabaceae	Houblon	Jenjel	Headache.	Leaves.	Raw: topical.	Flavonoids, polyphenols.	Sesquiterpenoids, diterpenoids, triterpenoids.		[36,76]
21	*Cucurbita maxima* Duchesne	Cucurbitaceae	Potiron	Elkaraea	Migraine.	Seeds.	Decoction: inhalation.	Phytosterols (cucurbitacin), vitamins (tocopherols and carotenoids), unsaturated fatty acids.	Mineral salts (zinc, selenium).		[31,36,77]
22	*Juniperus phoenicea* L.	Cupressaceae	Genévrier de Phénicie	Aaraar	Different neurological conditions.	Aerial parts, berries.	Decoction, infusion, tablet.	Essential oil, flavonoids, terpenoids, diterpenes (totarol), lignans, tannins.	Phenyl propanoids and furanone glycosides, sugars, fatty acids, organic acids, sterols.	Toxic in higher doses.	[5,57]
23	*Juniperus oxycedrus* L.	Cupressaceae	Genévrier cade	Ttaga	Different neurological conditions.	Aerial parts, berries.	Infusion, decoction, ext. essential oil, tablet.	Cade oil.	Essential oil, phenolic compounds, sesquiterpenes, cresol.	Cade oil is toxic in excessive amounts.	[5,78]
24	*Bituminaria bituminosa* (L.) C. H. Stirt.	Fabaceae	Herbe au bitume, Trèfle bitumineux	Adna, Menita	Epilepsy.	Whole plant.	Infusion.	Phenylpropanoids, coumarins, furanocoumarins, pterocarpans, flavonoids, isoflavones (daidzein and genistein), meroterpenes, sesquiterpenes.	Chalcones, phenols, phenolic cinnamates, phenylpropenes, sterols, terpenes, tocopherols, benzofurans, fatty acids.	Toxic in excessive amounts.	[66,79]
25	*Glycyrrhiza glabra* L.	Fabaceae	La réglisse	Ark-essous	Head problems, psychosis.	Roots.	Decoction: oral/topical.	Triterpene saponins (glycyrrhizin), isoflavones, phytosterols, coumarins, polysaccharides, asparagine.	Pectins, simple sugars, amino acids, mineral salts, essential oil, gum, protein, resin, volatile oils, tannins, glycosides.		[31,36,76]
26	*Lotus corniculatus* L.	Fabaceae	Lotier corniculé	Lotus el karni	Insomnia, depression, tranquilizer, neurological and psychological disorders.	Leaves, aerial parts.	Infusion, decoction.	Flavonoids, tannins.	Alkaloids, terpenes, fatty acids.		[63,66,80]
27	*Senna alexandrina* Mill.	Fabaceae	Le séné	Sena-mekki	Head problems, psychosis.	Leaves.	Decoction, topical/oral.	Sennosides A and B, dianthrones, anthrone.	Polyphenols.		[36,81,82]
28	*Trigonella foenum-graecum* L.	Fabaceae	Le fenugrec	Helba	Anxiety.	Seeds.	Infusion, raw, cataplasm.	Essential oil, alkaloids, flavonoids.	Saponins, proteins, vitamins, minerals, carbohydrates.		[34,36]
29	*Hypericum perforatum* L.	Hypericaceae	St. John’s worth, Millepertuis	Mesmoun, Berslouna	Epilepsy, anxiety, depression, neurosedative, nervousness.	Flower heads.	Infusion.	Phenolic compounds (hyperforin), naphthodianthrones (hypericin), proanthocyanins, essential oil.	Flavonoids, amentoflavones, carotenoids, catechic tannins.		[46,54]
30	*Crocus sativus* L.	Iridaceae	Safran cultivé	Zaafran	Memory and learning, insomnia, tranquilizer, neurodegenerative diseases, mild-to-moderate depression, anxiety, headache.	Stamen.	Infusion, powder, raw.	Essential oil, crocetin glucosides (crocins), carotenoids, vitamins (B1, B2).	Flavonoids.	Toxic at higher doses.	[5,54,71,72,78]
31	*Ajuga iva* (L.) Schreb	Lamiaceae	Bugle ivette	Chengoura	CNS diseases, memory, mental nervousness.	Whole plant, aerial parts, leaves, flowers, roots.	Infusion, decoction, powder, cataplasm.	Iridoids, diterpenes, phytoecdysone, caffeic acids.	Steroids, terpenoids, flavonoids, fatty acids.		[63,72,83]
32	*Lavandula stoechas* L.	Lamiaceae	Lavande papillon, lavande à toupet, lavande stéchade	Helhal	Anxiety, depression.	Leaves, stems, flowers.	Decoction, infusion, ext. essential oil.	Essential oil (terpenes, linalyl acetate, linalool, cineole, limonene), tannins, coumarins, flavonoids.	Triterpenes, alcohols, ketones, polyphenols.		[31]
33	*Lavandula officinalis* Chaix syn. *Lavandula angustifolia* Mill.	Lamiaceae	Lavande vraie, lavande à feuilles étroites	Khzama	Stress, anxiety, nervousness, depression, insomnia, tranquilizer.	Leaves, stems, flowers.	Decoction, infusion, ext. essential oil.	Essential oil (linalyl acetate, cineol, linalool, borneol), flavonoids, tannins, coumarins.	Polyphenols.		[31]
34	*Melissa officinalis* L.	Lamiaceae	Melisse	Melissa	Depression, stress, anxiety, nervousness, insomnia, nervous tonic.	Aerial parts.	Decoction, infusion, ext. essential oil, cataplasm, ointment.	Essential oil (citral, caryophyllene, linalool, citronellal), flavonoids, triterpenes, tannins.	Terpenoids, polyphenols.	The essential oil has moderate toxicity at higher doses.	[34,36,54,84]
35	*Mentha arvensis* L.	Lamiaceae	Menthe des champs	Naana	Head problems, anxiety, psychosis, insomnia.	Aerial parts.	Raw.	Menthol, menthone.	Isomenthone, neomenthol, limonene, methyl acetate, piperitone, beta-caryophyllene, alpha-pinene, beta-pinene, tannins, flavonoids.		[54,85]
36	*Mentha aquatica* L.	Lamiaceae	Menthe aquatique	Hbak elmaa	Anxiety, hypochondria.	Aerial part.	Raw.	Menthol, menthone.	Flavonoids (luteolin, menthoside), phenols, triterpenes.		[36,54]
37	*Mentha rotundifolia* (L.) Huds.	Lamiaceae	Menthe à feuilles rondes, Menthe du Nil	Timarssat	Pain, mental illnesses, removal of “curses” and protect from “evil spirits”.	Aerial part.	Decoction.	Menthol, menthone, pulegone.	Phenolic acids, flavonoids.		[86,87,88]
38	*Mentha pulegium* L.	Lamiaceae	Menthe pouliot	Fliou	Insomnia, headache, migraine.	Aerial part.	Decoction.	Pulegone, isopulegone, menthol.	Terpenes, tannins.	The essential oil is highly toxic.	[31,54]
39	*Mentha x piperita* L.	Lamiaceae	La Menthe	Naana	Tranquilizer, anxiety.	Aerial parts.	Infusion, capsules, essential oil: topical.	Essential oil (menthol, menthone), flavonoids (luteolin, menthoside).	Phenols, triterpenes.		[31]
40	*Ocimum basilicum* L.	Lamiaceae	Basilic	Hbak	Epilepsy, depression, anxiety, sleeping disorders, insomnia. mental exhaustion, migraine.	Leaves, flowering tops.	Decoction, powder, ext. essential oil.	Essential oil (linalool, methyl-chavicol, methyl cinnamate, cineol).	Terpenes.		[54,72,89,90]
41	*Origanum majorana* L.	Lamiaceae	Marjolaine	Merdakouche	Anxiety, insomnia, nervousness.	Aerial parts, ext. Essential oil.	Infusion.	Essential oil (sabinene hydrate, linalool, carvacrol), caffeic acid, rosmarinic acid, flavonoids.	Triterpenes.		[31]
42	*Origanum vulgare* L.	Lamiaceae	Origan	Zaatar	Anxiety, sleeping disorders.	Aerial parts, ext. essential oil.	Infusion.	Essential oil (thymol, carvacrol, borneol, linalool, beta-bisabolene, caryophyllene), flavonoids.	Phenolic acids, tannins.		[31]
43	*Rosmarinus officinalis* L.	Lamiaceae	Romarin	Leklil	Memory, slightly anti-depressive, psychical stimulant, nervousness.	Leaves, flowers, aerial parts.	Infusion, decoction, maceration.	Essential oil (borneol, camphor, camphene, cineol), flavonoids (apigenin, diosmin), rosmarinic acid, alkaloids(rosmaricin).	Tannins, diterpenes.	Toxic at higher doses.	[34,54,72]
44	*Salvia Officinalis* L.	Lamiaceae	Sauge	Miramia	Stress, tranquilizer, nervous tonic, nervousness, memory, insomnia, agitation in AD.	Leaves, stems.	Raw leaves, infusion, Decoction, maceration.	Essential oil, phenolic compounds (coumarins, flavonoids, tannins).	Alkaloids, carbohydrate, fatty acids, glycosidic derivatives, poly acetylenes, steroids, terpenoids.	Toxic in case of prolonged use or at high doses.	[34,54,72,91]
45	*Stachys arvensis* (L.) L.	Lamiaceae	Épiaire des champs	Chatra	Antidepressant, anxiety.	Capitulum.	Decoction.	Hydroxyflavone-allosylglucosides.	Flavonoids, iridoids, fatty acids, phenolic acids, diterpenoids.		[38,63,92,93]
46	*Stachys officinalis* L. Syn. *Betonica officinalis* L.	Lamiaceae	la Bétoine	Chatra	Nervous system stimulant, stress, insomnia, anxiety, tranquilizer, memory deficit, lightly sedative.	Aerial parts.	Tea of dried leaves, extract of flowers.	Stachydrine, betonicine, betaine, choline.	Iridoids, flavonoid, tannins, fatty acids, phenolic acids, diterpenoids, phenols, hydroxycinnamic acid derivatives, sesquiterpenes.		[54,92,93,94]
47	*Thymus vulgaris* L.	Lamiaceae	Thym	Zaatar	Anxiety, phobia.	Aerial parts, ext. essential oil.	Infusion, decoction.	Essential oil (thymol, carvacrol, linalool, cineol), flavonoids.	Phenolic acids, saponins, tannins.		[34,54,95,96]
48	*Cinnamomum camphora* (L.)	Lauraceae	Camphrier	Kafour	Migraine.	Wax.	Infusion: topical.	Camphor.	Essential oil (camphor, safrole, eugenol, terpineol), lignans.	Should be used externally only.	[31,36]
49	*Cinnamomum verum* J. Presl	Lauraceae	Cannelier	Karfa	Migraine.	Bark.	Raw: oral/topical.	Essential oil (cinnamaldehyde: 65–75%).	Polyphenols (vanillic acid, caffeic acid, gallic acid, p-coumaric acid, ferulic acid, cinnamic acid, proanthocyanidins A and B, kaempferol,), tannins (phlobatannins), coumarins, mucilages, eugenol.		[31]
50	*Lawsonia inermis* L. Syn. *Lawsonia alba*	Lythraceae	Henné	Henna	Anxiety, hypochondria.	Leaves	Raw: topical.	Naphtoquinones (lawsone).	Polyphenols, coumarins, flavonoids, tannins, alkaloids, terpenoids, sterols, carbohydrates, proteins, fatty acids.		[31]
51	*Punica granatum* L.	Lythraceae	Grenadier	Rommane	Headache.	Fruit peels, fruits.	Decoction: topical.	Flavonoids, ellagitannins, alcaloids.	Triterpens, polyphenols.	Peels may be toxic if consumed in excess.	[31,97]
52	*Myristica fragrans* Houtt.	Myristicaceae	Muscadier	Djouz-ettib	Head problems, psychosis.	Seeds.	Raw: topical.	Essential oil (α-pinene, β-pinene, alpha terpinene, beta terpinene, myristicin, elemicin, safrole).	Fixed oil (myristicin).	Toxic overdose may cause CNS excitation with anxiety/fear.	[98]
53	*Syzygium aromaticum* (L.) Merr. & L.M.Perry. syn. *Eugenia caryophyllata*	Myrtaceae	Girofle	K’rounfel	Mental asthenia, loss of memory, migraine.	Flower buds, leaves, stems.	Infusion, essential oil: topical.	Essential oil (eugenol, eugenol acetyl, methyl salicylate, pinene, vanillin).	Gum, tannins.		[54]
54	*Myrtus communis* L.	Myrtaceae	Le Myrte commun	Rihane	Anxiety, tranquilizer.	Leaves and flowers, essential oil, fruits may be eaten raw or dried.	Infusion, decoction.	Essential oil (alpha pinene, cineol, myrtenol), flavonoids, tannins.	Phenolic compounds.		[34,54]
55	*Peganum harmala* L.	Nitrariaceae	Harmel	Harmal	Anxiety, memory, nervous system disorders, Parkinson’s agitation.	Seeds, roots.	Infusion, decoction, fumigation.	Alkaloids (harmine, harmaline, harmalol).	Saponins, tannins, glycosides, terpenoids, steroids.	Toxic.	[34,54,99]
56	*Fraxinus angustifolia* Vahl.	Oleaceae	Frêne à feuilles étroites	Dardar	AD, different nervous system conditions.	Barks, leaves, grains.	Infusion.	Tannins, flavonoids, coumarins, essential oil, resin, malic acid.	Saccharides, minerals, vitamins.		[5,63,66,100]
57	*Olea europaea* L.	Oleaceae	Olive	Zitoun	Memory, anxiety, head problems, psychosis, depression.	Leaves, fruits, fruit oil.	Infusion, raw.	Oleuropein, oleuropeoside, monounsaturated fatty acids, tyrosol fatty acid esters, hydroxytyrosols, oleic acid, tocopherols.	Flavonoids, tannins, saponines, saccharides, triterpenes, vitamins, minerals.		[5,54]
58	*Papaver rhoeas* L.	Papaveraceae	Coquelicot	Khashkhash, thekouche	Insomnia, stress; tranquilizer, nervousness.	Flowers.	Infusion.	Alkaloids (papaverine, rheadine, isorheadine, rhoeagenine).	Anthocyanins, mucilage, tannins.	Toxic.	[54,72]
59	*Passiflora incarnata* L.	Passifloraceae	Passiflore	Nouar al saa	Epilepsy, depression, insomnia, anxiety, nervousness, hysteria, stress, tranquilizer.	Leaves, flowers.	Infusion, compressed.	Indole alkaloids (harmane), glycosidic flavonoids (vitexin, isovitexin, orientin), flavonoids (luteolin, chrysin, kaempferol, apigenin), glycosides (passiflorine), alkaloids (harmine, harmaline), palmitic acid, myristic acid.	Maltol, cyanogenetic glucosides (gynocardin).		[54,72,101]
60	*Pinus halepensis* Mill.	Pinaceae	Pin maritime	Snoubar, Z’koukou (fruits)	Memory deficits, tranquilizer, strengthen the nervous system.	Leaves, buds, bark seeds, resin.	Decoction, infusion, chewed, ext. ointment, cataplasm, powder, essential oil.	Essential oil.	Terpenoids, phenolic acids, flavonoids, fatty acids, steroids, aldehydes, ketones.		[34,66,72,102]
61	*Avena sativa* L.	Poaceae	Avoine	Chofan	Depression, asthenia, stress, insomnia, nervous fatigue, stimulates the nervous system.	Seeds, dried stems.	Infusion, tincture.	Saponins, alkaloids, trigonelline, silicic acid.	Proteins, vitamins B, minerals.		[54]
62	*Nigella sativa* L.	Ranunculaceae	Nigelle	Sanouj, Zrarâ	Anxiety.	Seeds.	Decoction, infusion, ext. powder, essential oil.	Fatty acids (linoleic acid, oleic acid), saponins, essential oil.	Alkaloids (Nigellimine N-oxide, nigellidine, nigellicine), carbohydrates, proteins, minerals, tannins.	Toxic (melanthin).	[34,54]
63	*Rhamnus alaternus* L.	Rhamnaceae	Le Nerprun alaterne	Melilesse	Neuroprotective.	Barks, leaves, fruits.	Powder, decoction, infusion.	Polyphenols, anthraquinones (emodin), anthrone, anthranols.	Tannins, anthocyanins, alkaloids.		[54,63,66,72,89,103]
64	*Zizyphus lotus* L.	Rhamnaceae	Le jujubier sauvage	Sedra	Neuroprotective, promoting memory and learning, insomnia, forgetfulness, hypnotic sedative, anxiolytic.	Leaves, fruits, roots.	Infusion, decoction, powder, fruits may be eaten raw or dried.	Tannins, vitamins, flavonoids, polyphenols, polysaccarides.	Alkaloids, minerals.		[54,57,72,104]
65	*Crataegus oxyacantha* L.	Rosaceae	Aubepine	Boukhrourou, Bou m’kherry	Epilepsy, loss of memory, insomnia, neurosedative, sleep disorders.	Flowers, fruits.	Infusion decoction, tincture, compressed.	Flavonoids (rutin, quercetin), flavones (vitexin, orientin, rhamnosylvitexin), triterpenes, proanthocyanes, polyphenols, tannins, coumarins, saponins, alkaloids (nicotine).	Amines, anthocyans, phenolic acids, triterpenic acids, sitosterols, purines.		[5,54,72]
66	*Crataegus azarolus* L.	Rosaceae	Azerolier	Zaarour	Anxiety, stress, psychical disorders, insomnia, nervous tonic.	Leaves, flowers, fruits.	Extracts, tincture, fruit can be eaten raw, cooked, or as preserves.	Essential oil, tannins, amino acids, proanthocyanidins, flavonoids.	Sugars (fructose, glucose et rhamnose), vitamins.		[5,72,89,100]
67	*Eriobotrya japonica* (Thunb.) Lindl	Rosaceae	Le néflier du Japon	Nifla	Headache, dizziness.	Leaves	Decoction	Quercetin, ursolic acid, oleanolic acid, tannins, chlorogenic acid, caffeoylquinic acid.	Polyphenols, flavonoids, carotenoids, triterpenoids.		[105,106]
68	Rosa canina L.	Rosaceae	Églantier	Nasrine, Ouardzeroub	Tranquilizer, anxiety, depression.	Fruits (rose hips), leaves, flowers.	Tea infusion.	Vitamins (C, A, B1, B2, P, K), flavonoids, tannins, citric acid, carotenoids, essential oil, D-sorbitol.	Polyphenols, carotenoids, carbohydrates, fatty acids.		[54,72,89]
69	*Galium verum* L.	Rubiaceae	Caille-lait	Fouaoua	Epilepsy.	Aerial parts, flowers.	Infusion.	Iridoids (asperulosides), flavonoids, anthraquinones, alcanes.	Phenolic compounds, tannins, saponins, triterpenes, essential oil, wax, pigments, vitamin C.		[54,66]
70	*Citrus limon* (L.) Osbeck.	Rutaceae	Citronnier	Laymoune	Dizziness.	Fruits.	Decoction.	Essential oil, terpenes (limonene), flavonoids (hesperidin), vitamins, mucilage.	Sesquiterpenes, aldehydes (citral), coumarins.		[31,36]
71	*Ruta chalepensis* L.	Rutaceae	Rue de Chalep	Fidjel	Headache, mental disorders.	Aerial parts	Decoction	Coumarins (furanocoumarins, dihydrofuranocoumarins), furoquinoline alkaloids.	Flavonoids, essential oil.		[107,108]
72	*Populus Nigra* L.	Salicaceae	Peuplier noir	Safsaf	Neurodegenerative disorders.	Leaves, flower buds, barks.	Infusion, tincture, powder, ointment.	Tannins, flavonoids, saccharides, essential oil.	Phenolic compounds, terpenoids.		[5,73,109]
73	*Santalum album* L.	Santalaceae	Santal blanc	Sandel	Migraine.	Bark, fruits.	Decoction: topical/oral.	Essential oil, tannins.	Resin.		[31]
74	*Atropa belladonna* L.	Solanaceae	Belladone	Sitt Elhossn	Parkinson’s disease, neurological disorders, tranquilizer.	Leaves, roots.	Tincture, decoction.	Tropane alkaloids.	Nicotine, flavonoids, coumarins.	Toxic (effects includes pupil dilatation, confusion, hallucination).	[34,54,72]
75	*Datura stramonium* L.	Solanaceae	Stramoine	Tatura, Mendj	Parkinson’s disease, stress.	Leaves, capitulum, seeds.	Tincture, decoction, fumigation, powder.	Tropane alkaloids (hyoscyamine, hyoscine).	Flavonoids, withanolides, coumarins, tannins, minerals.	Highly toxic.	[54,72]
76	*Tamarix aphylla* (L.) H.Karst	Tamaricaceae	Tamarix aphylla	Tahtah	Headache.	Leaves.	Decoction.	Flavonoids, polyphenols, tannins.	Catechins, triterpenoids.		[110,111]
77	*Tilia cordata* Mill.	Tiliaceae	Tilleul	Zayzafoune	Epilepsy, neurosedative, sleep disorders, stress, anxiety.	Flowers.	Infusion.	Flavonoids (quercetin, rhamnoside, kaempferol).	Tannins, essential oil, mucilage.		[54,72,89]
78	*Valeriana tuberosa* L.	Valerianaceae	Valériane	Senbel, nardine	Epilepsy, stress, anxiety, insomnia.	Roots and rhizome.	Infusion, decoction.	Essential oil (bornyl acetate, beta-caryophyllene), iridoids (valepotriate), alkaloids	Tannins, flavonoids.		[5,46]
79	*Valeriana officinalis* L.	Valerianaceae	Valériane	Senbel	Epilepsy, stress, anxiety, insomnia, nervousness.	Roots and rhizome.	Infusion, decoction, tincture, powder, compressed.	Essential oil (bornyl acetate, beta-caryophyllene), iridoids (valepotriate), alkaloids.	Tannins, flavonoids.		[5,54,72,90]
80	*Verbena officinalis* L.	Verbenaceae	Verveine	Louisa	Tranquilizer, lightly sedative, anxiolytic, anticonvulsant, lightly antidepressive, insomnia, anxiety, mental fatigue.	Aerial parts.	Tincture, infusion, powder.	Iridoids (verbenone, verbenaline), Essential oil.	Mucilage, tannins, flavonoids, phenolic acids.		[54,112,113,114]
81	*Zingiber officinale* L.	Zigiberaceae	Gingembre	Zanjabil	Anxiety, head problems, psychosis.	Rhizome.	Infusion, maceration, capsules, tincture, ext. essential oil.	Essential oil, sesquiterpenes, oleoresin, phenols (gingerol).	Phenolic compounds.		[34,54]
82	*Curcuma longa* L.	Zigiberaceae	Curcuma	Korkoum	Anxiety, hypochondria.	Rhizome.	Decoction, powder, cataplasm, tincture.	Essential oil, zingiberene, turmerone, curcuminoids (curcumin), resin.	Phenolic compounds, caffeic acid derivatives.		[31,34,36]

## Data Availability

Data are contained within the article.
